# Chemosensory genes in the antennal transcriptome of two syrphid species,*Episyrphus balteatus*and*Eupeodes corollae* (Diptera: Syrphidae)

**DOI:** 10.1186/s12864-017-3939-4

**Published:** 2017-08-07

**Authors:** Bing Wang, Yang Liu, Gui-Rong Wang

**Affiliations:** State Key Laboratory for Biology of Plant Diseases and Insect Pests, Institute of Plant Protection, Chinese Academy of Agricultural Sciences, Beijing, 100193 China

**Keywords:** *Episyrphus balteatus*, *Eupeodes corollae*, Transcriptome, Chemosensory genes, Odorant receptors, Syrphid olfaction

## Abstract

**Background:**

Predatory syrphid larvae are an important natural enemy of aphids in cotton agro-ecosystems in China. Their behaviors in prey foraging, localization and oviposition greatly rely on the perception of chemical cues. As a first step to better understand syrphid olfaction at the molecular level, we have performed a systematic identification of their major chemosensory genes.

**Results:**

Male and female antennal transcriptomes of *Episyrphus balteatus* and *Eupeodes corollae* were sequenced and assembled using Illumina HiSeq2000 technology. A total of 154 chemosensory genes in *E. balteatus* transcriptome, including candidate 51 odorant receptors (ORs), 32 ionotropic receptors (IRs), 14 gustatory receptors (GRs), 49 odorant-binding proteins (OBPs), 6 chemosensory proteins (CSPs) and 2 sensory neuron membrane proteins (SNMPs) were identified. In *E. corollae* transcriptome, we identified 134 genes including 42 ORs, 23 IRs, 16 GRs, 44 OBPs, 7 CSPs and 2 SNMPs. We have provided full-length sequences of the highly conserved co-receptor Orco, IR8a/25a family and carbon dioxide gustatory receptor in both syrphid species. The expression of candidate OR genes in the two syrphid species was evaluated by semi-quantitative reverse transcription PCR. There were no significant differences of transcript abundances in the respective male and female antenna, which is consistent with differentially expressed genes (DEGs) analysis using the FPKM value. The sequences of candidate chemosensory genes were confirmed and phylogenetic analysis was performed.

**Conclusions:**

This research comprehensively analyzed and identified many novel candidate chemosensory genes regarding syrphid olfaction. It provides an opportunity for understanding how syrphid insects use chemical cues to conduct their behaviors among tritrophic interactions of plants, herbivorous insects, and natural enemies in agricultural ecosystems.

**Electronic supplementary material:**

The online version of this article (doi:10.1186/s12864-017-3939-4) contains supplementary material, which is available to authorized users.

## Background

Natural enemies are important in controlling the population of insect pests in agricultural ecosystems [1–6]. The presence of natural enemies is detected through chemical cues emitted by prey and host plants, alone or in association, and received by the antennal sensory system in the multi-trophic environment to result in various behavioral choices including prey forage, localization, oviposition and escape [7–17]. Recently the study of insect peripheral sensory system and chemosignal transduction has experienced considerable progress due to the development of bioinformatics-based approaches and protein function prediction methods [18]. In particular, a large amount of information is provided by antennal transcriptome projects from various insect taxa [19–28].

In general, the process of chemoreception, including olfaction and taste, involves several families of genes, including odorant receptors (ORs), ionotropic receptors (IRs), and gustatory receptors (GRs) [20, 29–31]. In addition, odorant binding proteins (OBPs), chemosensory proteins (CSPs) and sensory neuron membrane proteins (SNMPs) also play crucial roles in chemoreception [32–36]. The insect chemoreceptor superfamily including ORs and GRs was first identified in the *Drosophila melanogaster* genome [18, 21]. Insect odorant and gustatory receptors were once thought to be G-protein-coupled receptors just like ORs in worms and vertebrates, but subsequent studies have shown a lack of homology to vertebrate ORs [37]. One such superfamily encoding ORs is highly divergent across insect taxa with sequences and frequencies varying to a large extent [18, 38, 39]. ORs are broadly tuned to alcohols, ketones, and esters generally present in the environment [40, 41]. Another family encoding GRs, or receptors for taste or contact stimuli, is also very divergent across insect taxa [31]. On the contrary, one example of exceptionally conserved GRs are GR21a and GR63a, which work together as a CO_2_ receptor in *Drosophila* [42]. Such chemoreceptors play an important role in host seeking behaviors in many insects but, especially seen in mosquitoes [43, 44]. A new insect chemosensory family was identified recently and given the name ionotropic receptors (IRs). These IRs belong to the ionotropic glutamate receptor superfamily (iGluRs) and were identified in both the olfactory and gustatory systems [30, 45]. IRs are more greatly conserved than ORs and GRs but considerable variations can be observed in ligand-binding domains. They are mainly tuned to acids, amines and other odorants that are not detected by ORs [30, 45, 46].

Since chemosensory gene families were characterized in two important model species, *D. melanogaster* and *Anopheles gambiae* [47, 48], a growing number of chemosensory genes have been identified from many Dipteran species, such as *Musca domestica* [49], *Bactrocera dorsalis* (genome: assembly ASM78921v2), *Calliphora stygia* [50], *Glossina morsitans morsitans* [51], and *Mayetiola destructor* Say [20]. Protein prediction methods have been the first step for functional identification of chemosensory genes. All information regarding insect chemosensory was obtained bioinformatically and has been beneficial in understanding insect processing of diverse volatile compounds and cross-species differences in chemical communication.

The syrphids belong within the Diptera order and their larvae are aphid-specific natural enemies [1, 2, 4, 5, 8]. Due to the larvae’s agricultural importance via potential applications, several reports have been published on the chemical ecology of these insects. In *Episyrphus balteatus* DeGeer, larvae may use a sesquiterpene as a kairomone [17, 52] and other potential semiochemicals to locate their prey [8, 53]. In *Sphaerophoria rueppellii*, adult females are strongly attracted to odors from aphid colonies showing that specific volatile compounds are important to detect their prey [1]. Some studies on the relationships between aphid or host plant volatile emissions and aphid localization and foraging behavior have shown strong associations with syrphid recognition. One striking finding has shown that volatiles from plants attacked by aphids produce strong electrophysiological responses from the antennae of syrphids [7, 8]. These studies indicate that detecting prey-derived volatiles ((E)-β-farnesene), herbivore-induced plant volatiles (monoterpenes and sesquiterpenes), or naturally occurring general leaf volatiles (GLVs; alcohols, aldehydes and esters) help natural enemies to select oviposition sites and locate their prey [8, 12, 13, 17, 52–55].

Despite these reports on chemosensory behavior, little is known on the molecular basis of syrphid olfaction. Therefore, the identification of predatory syrphid chemosensory gene families will help reveal how syrphids forage on their prey and choose oviposition sites. In this study we selected two syrphid species, *E. balteatus* and *Eupeodes corollae* Fabricius, active in northern China cotton fields to perform antennal transcriptome sequencing in order to explore and compare chemosensory genes in the two species. A total of 154 and 134 chemosensory candidate genes were identified in *E. balteatus* and *E. corollae* transcriptomes, respectively, including ORs, IRs, GRs, OBPs, CSPs and SNMPs. Furthermore, we report the expression profile of the OR families found in each insect transcriptome. A comparison between these two syrphids and other insect species revealed candidate chemosensory genes that could be involved in prey selection and plant volatile recognition. The discovery of putative chemosensory genes gives way for further exploration into functional assessments regarding chemoreception association.

## Results

### Antennal transcriptome sequencing and sequence assembly

*E. balteatus* and *E. corollae* antennal transcriptomes were sequenced using the Illumina HiSeq 2000 platform combined with Trinity assembly. Approximately 68.71 million and 77.28 million raw-reads were obtained from *E. balteatus* male and female antenna, respectively, reduced after filtering, to 65.69 and 74.25 million clean-reads. These were assembled into 57,950 unigenes for male and 68,165 for female. A final transcript dataset with 53,575 unigenes was obtained, consisting of 17,407 distinct clusters and 36,168 distinct singletons. The dataset was 47.61 megabase (Mb) in size with a mean length of 889 bp and N50 of 1724 bp (Additional file 1: Table S1). Parallel experiments generated 80.15 million and 77.38 million raw-reads in *E. corollae* male and female, respectively, and 65.69 and 74.25 million clean-reads. From these datas 54,116 and 61,220 unigenes were obtained for male and female, respectively. The final transcript dataset of *E. corollae* contained 50,942 unigenes with a mean length of 1039 bp and N50 length of 2104 bp, consisting of 18,054 distinct clusters and 32,888 distinct singletons (Additional file 1: Table S1). In addition, unigenes with a sequence length > 500 bp accounted for 47.64% and 43.59% of the *E. corollae* and *E. balteatus* transcriptome assembly, respectively.

### Homology analysis and gene ontology (GO) annotation

A BLASTX homology search against the NCBI non-redundant protein database indicated 23,680 (44.2%) and 25,606 (50.3%) unigenes from *E. balteatus* and *E. corollae*, respectively, with sequence similarities to known proteins using a cut-off E-value of 10^−5^. For *E. balteatus*, the larger number of similar genes (27.4%) belonged to *Ceratitis capitata* followed by *M. domestica* (20.4%), *D. melanogaster* (7.4%), *Drosophila willistoni* (3.1%), *Drosophila virilis* (3.0%) and *Drosophila mojavensis* (2.5%). For *E. corollae*, again *C. capitata* was best represented (25.1%), followed by *M. domestica* (18.8%), *D. melanogaster* (7.1%), *D. willistoni* (2.9%), *Acyrthosiphon pisum* (2.8%), and *D. virilis* (2.5%) (Additional file 2:Fig. S1A).

Gene ontology (GO) annotations were used to classify the transcripts into functional groups in accordance with specific GO categories. A total of 12,441 (23.22%) of all predicted proteins from *E. balteatus* and 12,425 (24.39%) predicted proteins from *E. corollae* were assigned to at least one GO term (Additional file 2: Fig. S1B). The GO terms distribution in the three categories were similar in the two species. In the “molecular function” category, the most abundant GO terms were “binding” and “catalytic activity”. In the “biological process” category, “cellular process”, “single-organism process” and “metabolic process” were the most represented. Finally, “cell”, “cell part”, and “organelle” were the most abundant GO terms in “cellular component” category (Additional file 2: Fig. S1B). GO terms associated with chemosensory genes were distributed in the “biological process” category (e.g. “cellular process”, “developmental process”, “response to stimulus”, “establishment of localization”, and “biological regulation”, etc.), “molecular function” category (e.g. “molecular transducer activity”, etc.) and “cellular component” category (e.g. “extracellular region”, “membrane part”, “membrane”, etc.).

### Candidate ORs in *E. balteatus* and *E. corollae*

Based on our analysis of the antennal transcriptomes in the two species, 51 and 42 transcripts for candidate ORs were identified in the combined male and female data sets from *E. balteatus* and *E. corollae*, respectively (Additional file 3: Table S2). A total of 21 *E. balteatus* ORs (EbalORs) and 29 *E. corollae* ORs (EcorORs) contained full-length open reading frames (ORFs), whose translation products are predicted to possess 2–8 transmembrane domains (TMDs). Other partial length transcripts encoded proteins exhibiting overlapping regions with low identity and were classified as unique genes. After a more exhaustive comparison with OR genes from other insect species, we found that all putative EbalORs shared between 22% and 86% amino acid identity with other ORs, with almost identical values (22% to 87%) for EcorORs. Detailed information is reported in Additional file 3 and Table S2.

**Fig. 1:**
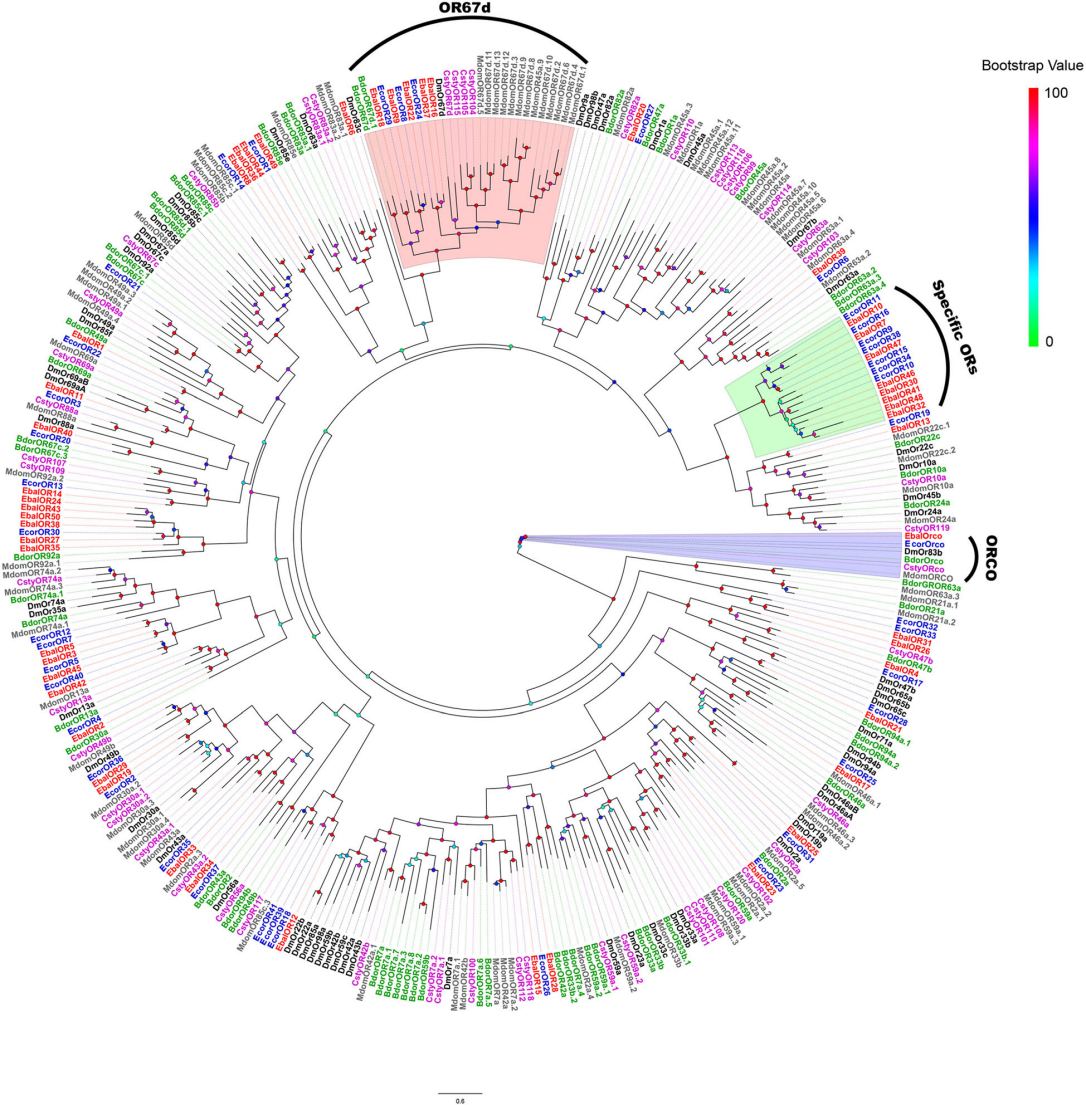
Phylogenetic tree of candidate *E. balteatus* and *E. corollae* ORs and other Dipteran ORs. The distance tree was rooted by the conservative Orco gene orthologous. Bootstrap values are shown. The Orco clade, OR67d clade and specific EbalORs and EcorORs clade are shown. Species in this phylogeny include *E. balteatus* (Ebal, *red*), *E. corollae* (Ecor, *blue*), *Drosophila melanogaster* (Dm, *dark*), *Bactrocera dorsalis* (Bdor, *green*), *Calliphora stygia* (Csty, *magenta*), and *Musca domestica* (Mdom, *gray*)

We next performed a phylogenetic analysis using our candidate ORs and the ORs from four other Diptera species including *B. dorsalis*, *C. stygia*, *D. melanogaster* and *M. domestica* (Fig. 1). Clustered with DmOR83b, the highly conserved co-receptor Orco, orthologous genes were identified in the antennal transcriptomes of both syrphid species, and named EbalOrco and EcorOrco. As expected, sequence identity between EbalOrco and EcorOrco is very high (97.27%). Among the other ORs, five EbalORs (EbalOR9, 16, 18, 22 and 37) and three EcorORs (EcorOR8, 24 and 29) clustered with DmelOR67d, the pheromone receptor from *D. melanogaster*. This OR67d specific clade also included the OR67d orthologues from *M. domestica* and *B. dorsalis*. Two of these genes, EbalOR16 and EcorOR24, are full-length transcripts with 71.65% amino acid identity. The remaining ORs in this group were highly divergent among different species. Within the Dipteran OR sequences, we found a species-specific clade including eight members from *E. balteatus* (EbalOR7, 10, 30, 32, 41, 46, 47 and 48) and seven from *E. corollae* (EcorOR9, 10, 11, 15, 16, 34 and 38) that shared low identities with other Dipteran ORs (Fig. 1).

Amino acid identity between gene products correlates with similarity between genes. An amino acid sequence comparison between EbalORs and EcorORs revealed 33 pairs of orthologous ORs (including Orco) with 73.86% identity and the amino acid identity of complete OR ORFs was 84.00%. Sequence similarity percentages of the 28 pairs of homologous ORs are greater than 60% (Additional file 4: Table S3). In addition to the Orco family, orthologous groups with identities higher than 90% include the EbalOR29/EcorOR36, EbalOR3/EcorOR5, EbalOR13/EcorOR19 EcorOR22 with/EcorOR22 with 94.88%, 92.20%, 91.46% and 91.30% sequence identities, respectively. In addition, 10 EbalOR sequences (EbalOR2, 5, 7, 8, 11, 14, 21, 23, 25 and 33) are closely related to EcorOR homologues (EcorOR3, 4, 7, 9, 13, 14, 23, 28, 31 and 35) with identities greater than 80% (Additional file 4: Table S3; Fig. 1). All of these highly homologous proteins may play important roles in olfactory recognition.

### Candidate GRs in *E. balteatus* and *E. corollae*

**Fig. 2:**
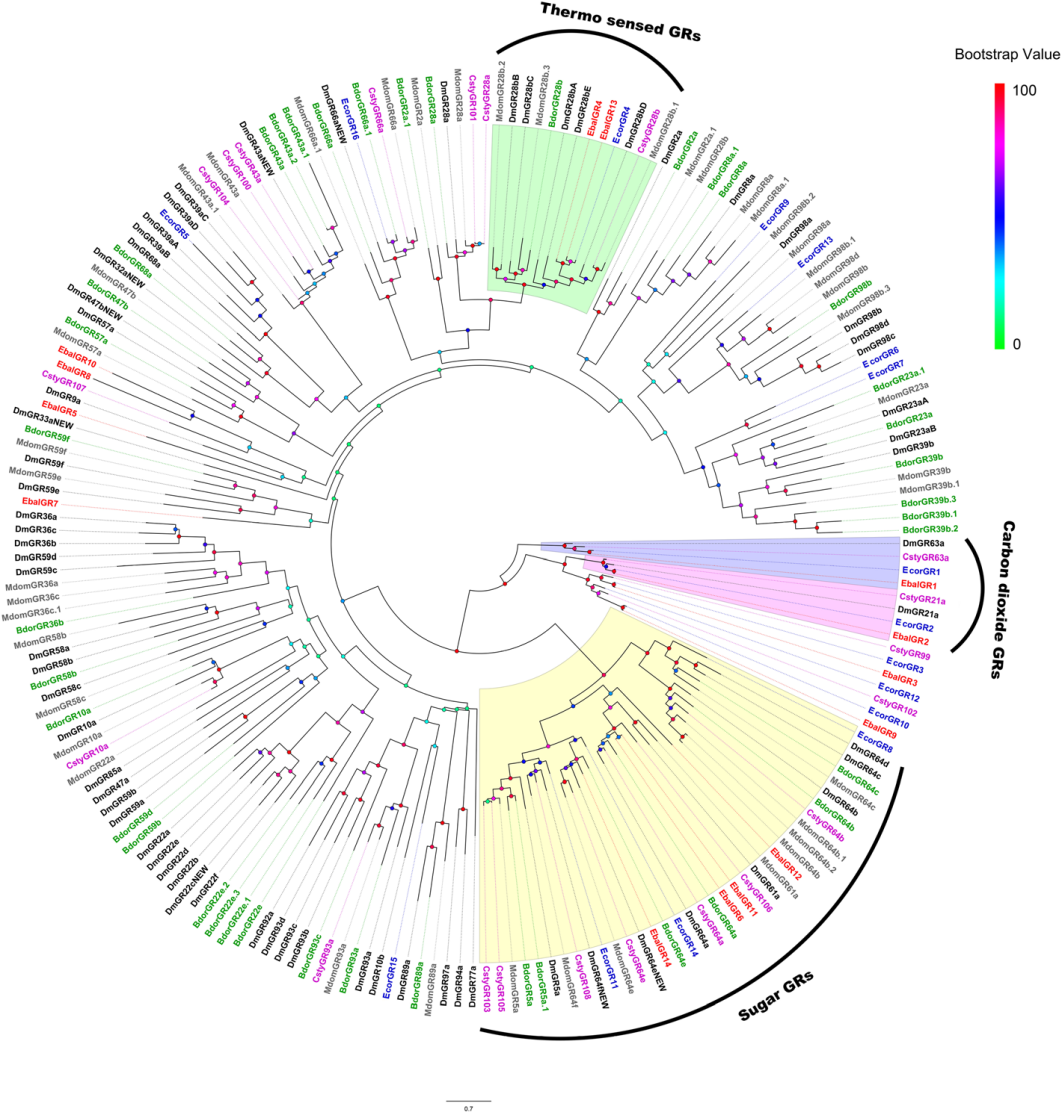
Phylogenetic tree of candidate *E. balteatus* and *E. corollae* GRs and other Dipteran GRs. The distance tree was rooted by the conservative carbon dioxide GRs gene orthologous. Bootstrap values are shown. The carbon dioxide GRs clade, thermos-sensed GRs clade and sugar GRs clade are shown. This tree was constructed using the species *E. balteatus* (Ebal, *red*), *E. corollae* (Ecor, *blue*), *D. melanogaster* (Dm, *dark*), *B. dorsalis* (Bdor, *green*), *C. stygia* (Csty, *magenta*), and *M. domestica* (Mdom, *gray*)

We have identified 14 and 16 candidate GR genes from *E. balteatus* and *E. corollae* transcriptomes, respectively (Additional file 3: Table S2). The majority of candidate EbalGRs and EcorGRs were partial fragments, with only three from *E. balteatus* and six from *E. corollae* encoding full-length proteins. These complete sequences all show six or seven TMDs with an intracellular N-terminus and extracellular C-terminus. Phylogenetic analysis with GRs from six Dipteran species suggest that *Drosophila* GR21a and GR63a, reported as carbon dioxide sensors [42, 56], clustered first with EbalGR2 and EcorGR2 and second with EbalGR1 and EcorGR1. In addition, EbalGR4, EbalGR13 and EcorGR4 showed high identities to thermoreceptor DmelGR28b responsible for rapid warmth avoidance [57]. Several other GRs clustered with members of candidate sugar detection GR (GR5a, GR61a and GR64a-f) sub-family (Fig. 2) [58–62].

### Candidate IRs in *E. balteatus* and *E. corollae*

**Fig. 3:**
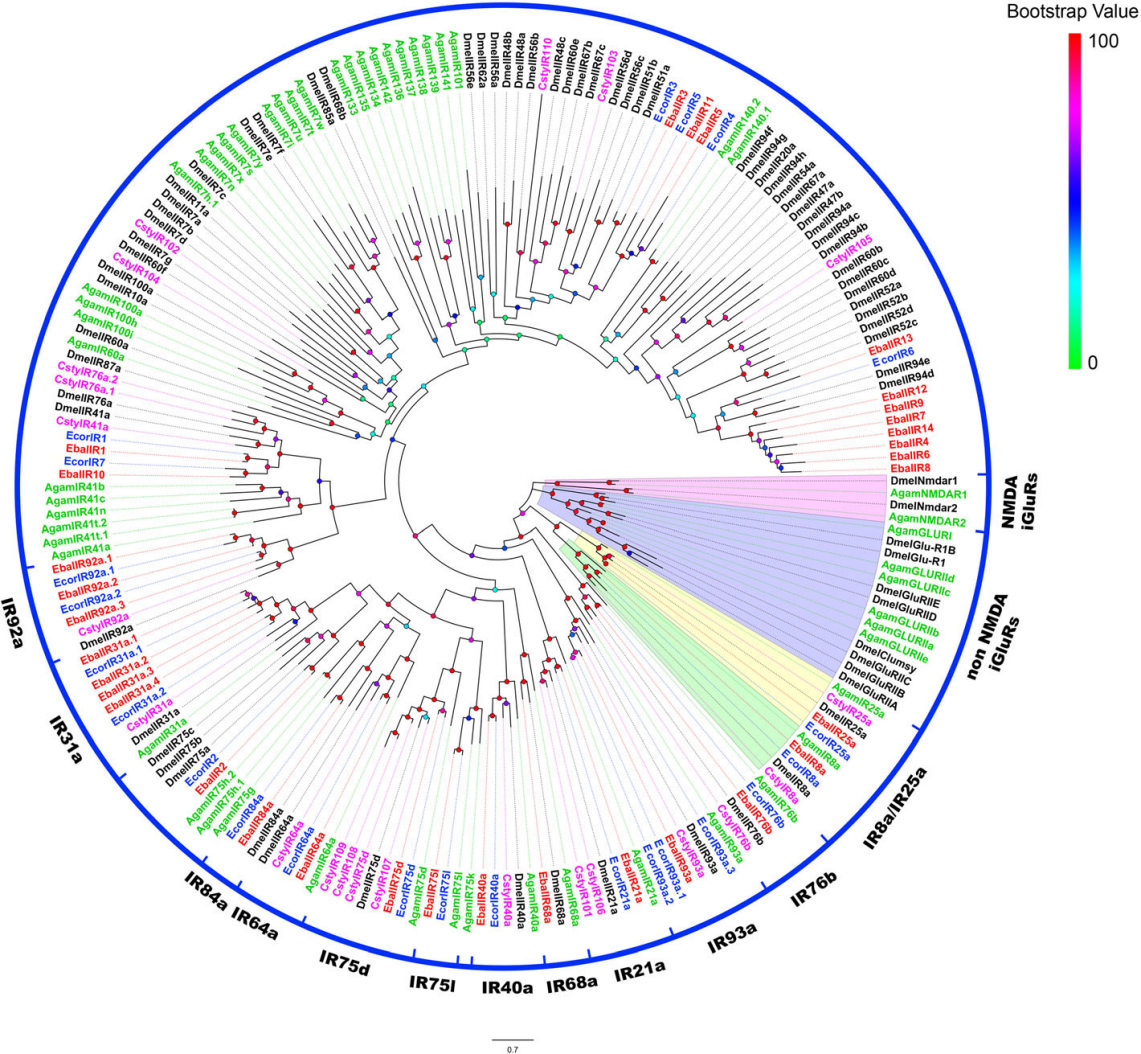
Phylogenetic tree of candidate *E. balteatus* and *E. corollae* IRs and other Dipteran IRs. The distance tree was rooted by the conservative IR25a/IR8a gene orthologues. Bootstrap values are shown. The IR25a/IR8a clade, iGluRs clade and some antennal-associated orthologue clade are shown. This tree was constructed using the following species: *E. balteatus* (Ebal, *red*), *E. corollae* (Ecor, *blue*), *D. melanogaster* (Dmel, *dark*), *Anopheles gambiae* (Agam, *green*), and *C. stygia* (Csty, *magenta*)

We identified 32 transcripts for putative ionotropic receptors in *E. balteatus* and 23 in *E. corollae*. Of these, seven EbalIRs and 14 EcorIRs contained full-length ORFs, with two to five TMDs (Additional file 3: Table S2). Among these we found the common conserved co-receptors IR8a (EbalIR8a and EcorIR8a) and IR25a (EbalIR25a and EcorIR25a) in both species. Other candidate IRs were found as partial sequences (Fig. 3).

In order to further distinguish putative IRs from iGluRs, all EbalIRs and EcorIRs were aligned with IRs from *A. gambiae*, *C. stygia* and *D. melanogaster*, as well as some AgamiGluRs and DmeliGluRs for phylogenetic analysis. The results showed that the candidate EbalIRs and EcorIRs clustered with presumed “antennal” orthologues IR76b, IR93a, IR21a, IR68a, IR40a, IR75l, IR75d, IR64a, IR84a, IR31a and IR92a, and were well separated from the AgamiGluRs and DmeliGluRs clade (Fig. 3) [63]. Interestingly, the conserved “antennal” orthologues, IR60a, was lacking from *E. balteatus* and *E. corollae* transcriptome assemblies, while IR68a was only absent from *E. corollae.* The sequences of *E. balteatus* clustering with DmelIR94d and DmelIR94e were quite divergent (Fig. 3; Additional file 5: Fig. S2). When compared to the orthologues within other species, these IRs may play different roles in olfaction.

### Candidate OBPs in *E. balteatus* an*d E. corollae*

We identified 49 different transcripts encoding candidate OBPs in *E. balteatus* and 44 in *E. corollae*, numbers similar to the 52 OBPs of *D. melanogaster* [64]. Of these, 38 transcripts of EbalOBPs and 31 EcorOBPs contained full-length ORFs with predicted signal peptide sequences (with EbalOBP31 as the only exception) (Additional file 3: Table S2).

**Fig. 4:**
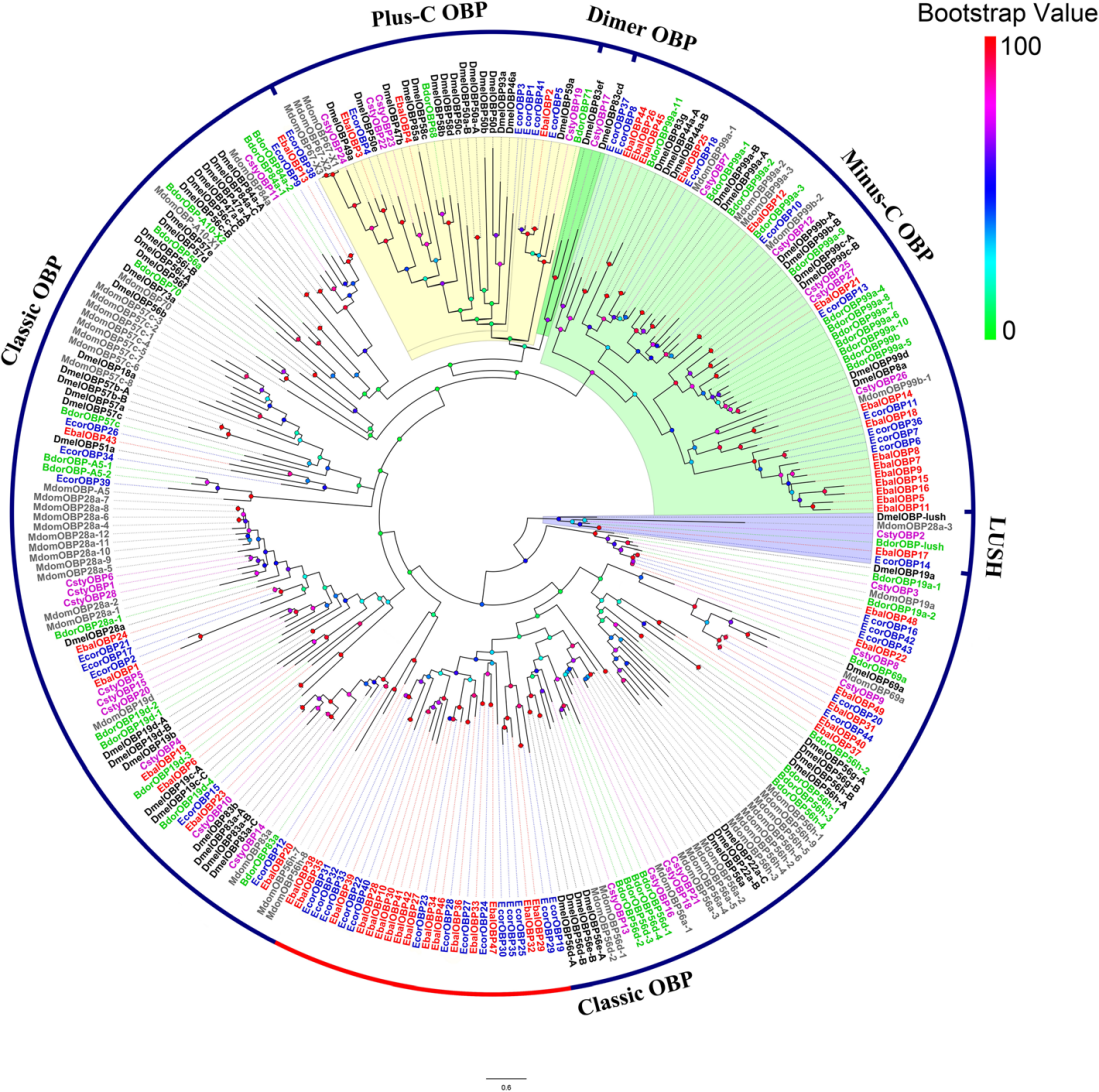
Phylogenetic tree of candidate *E. balteatus* and *E. corollae* OBPs and other Dipteran OBPs. The distance tree was rooted by lush gene orthologous. Bootstrap values are shown. The classic OBPs clade, Plus-C OBPs clade and Minus-C OBPs clade are shown. The species used to construct tree include *E. balteatus* (Ebal, *red*), *E. corollae* (Ecor, *blue*), *D. melanogaster* (Dmel, *dark*), *B. dorsalis* (Bdor, *green*), *C. stygia* (Csty, *magenta*), and *M. domestica* (Mdom, *gray*)

A phylogenetic tree was built with these sequences and those of orthologous from *B. dorsalis*, *C. stygia*, *D. melanogaster* and *M. domestica*. Among EbalOBPs, thirty-one showed the classic motif of six conserved cysteines, three were Plus-C (EbalOBP2, 3, 4) and fifteen were Minus-C (EbalOBP5, 7, 8, 9, 11, 12, 14, 15, 16, 18, 21, 25, 26, 44 and 45) (Fig. 4). For EcorOBPs, we found 30 classic, 5 Plus-C (EcorOBP1, 3, 4, 5, 41) and 9 Minus-C (EcorOBP6, 7, 8, 10, 11, 13, 18, 36 and 37) (Fig. 4) [64, 65]. One large group of classic OBPs, including 15 EbalOBPs and 14 EcorOBPs showed large differences compared to sequences of other species and could represent OBPs specific of syrphids (Fig. 4; Additional file 6: Fig. S3). We found the orthologue of DmelOBP-lush in both species, EbalOBP17 and EcorOBP14. These two proteins are 98.64% identical at the amino acid level between each other and 39.87% and 40.52% identity to DmelOBP-lush.

### Candidate CSPs in *E. balteatus* and *E. corollae*

**Fig. 5:**
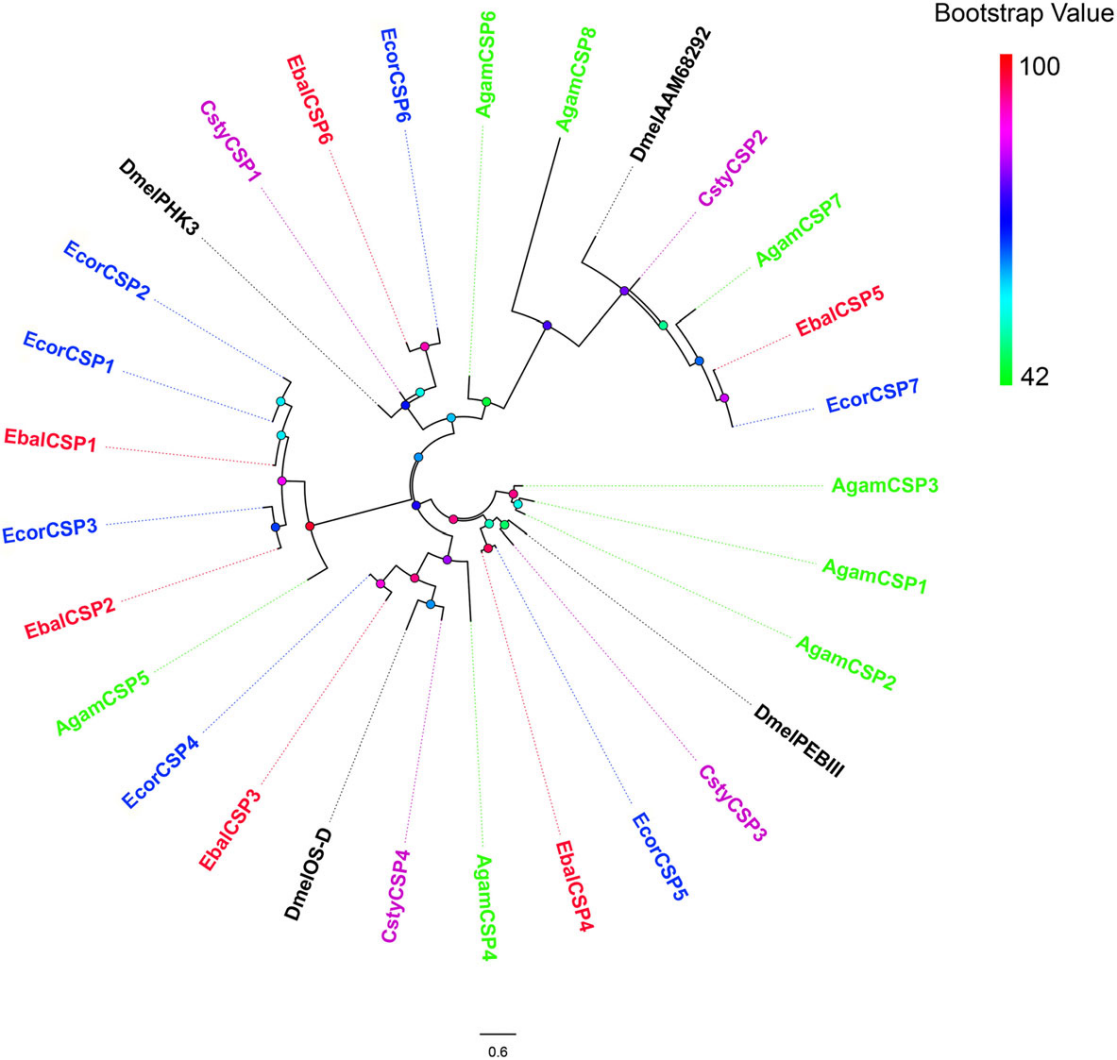
Phylogenetic tree of candidate *E. balteatus* and *E. corollae* CSPs and other Dipteran CSPs. The distance tree was rooted by AgamCSP1/2/3 genes. Bootstrap values are shown. The species used to construct tree include *E. balteatus* (Ebal, *red*), *E. corollae* (Ecor, *blue*), *D. melanogaster* (Dmel, *dark*), *A. gambiae* (Agam, *green*), and *C. stygia* (Csty, *magenta*)

Through bioinformatic analysis, six and seven different transcripts encoding candidate CSPs were identified from *E. balteatus* and *E. corollae* transcriptomes, respectively. Five EbalCSPs and six EcorCSPs represented full-length proteins and only EbalCSP6 lacked a signal peptide (Additional file 3: Table S2). All of the identified amino acid sequences possessed the highly conserved four-cysteine profile. A phylogenetic tree was built with all the syrphid CSPs and those of *A. gambiae*, *C. stygia*, *D. melanogaster* (Fig. 5).

### Candidate SNMPs in *E. balteatus* an*d E. corollae*

**Fig. 6:**
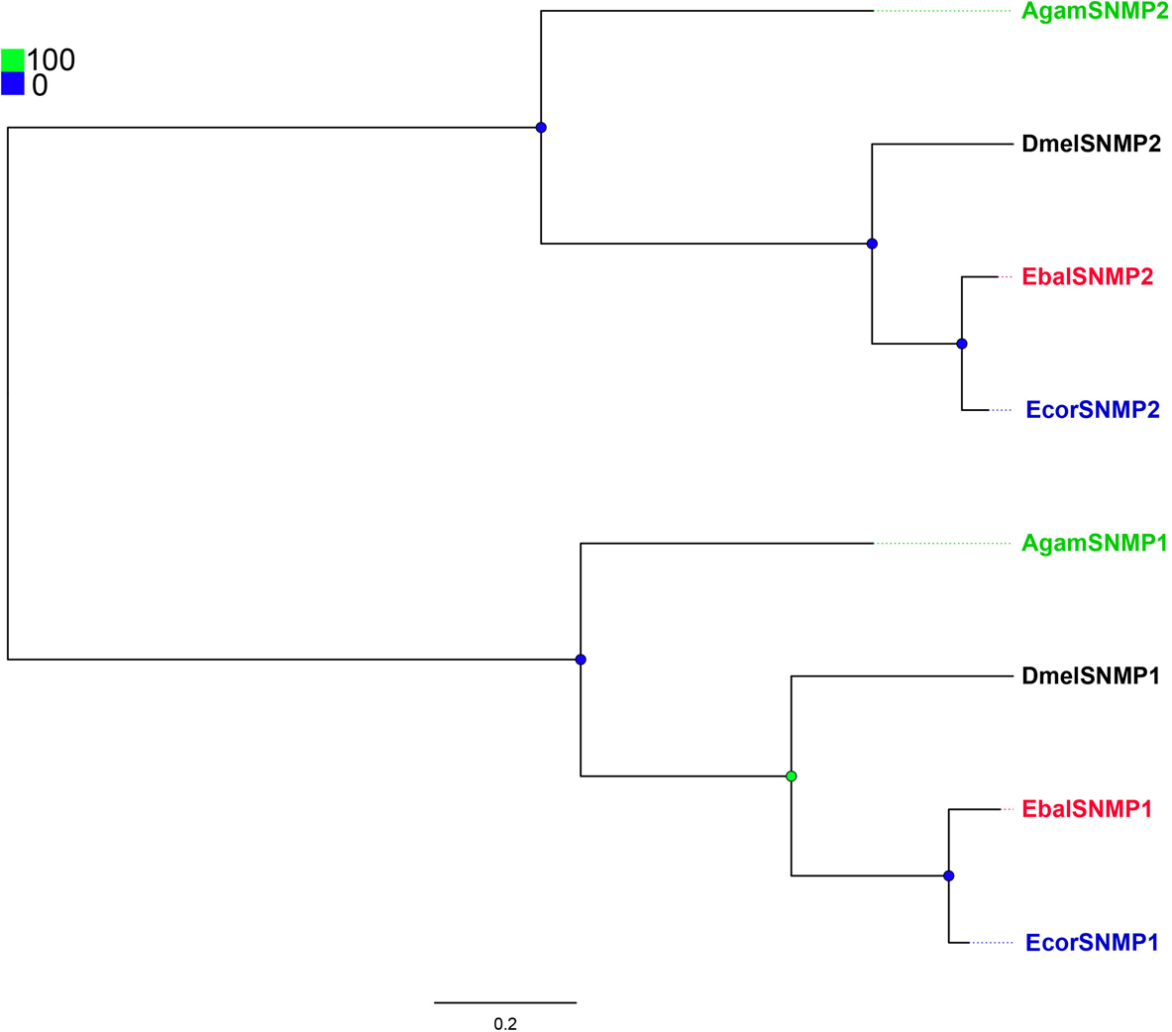
Phylogenetic tree of candidate *E. balteatus* and *E. corollae* SNMP and other Dipteran SNMP. Bootstrap values are shown. The species used to construct tree including *E. balteatus* (Ebal, *red*), *E. corollae* (Ecor, *blue*), *D. melanogaster* (Dmel, *dark*) and *A. gambiae* (Agam, *green*)

In both species, two SNMPs with full-length ORFs were identified possessing two TMDs (with EbalSNMP1 having a single TMD as an exception) (Additional file 3: Table S2). EbalSNMP1 and EcorSNMP1 are very similar to DmelSNMP1, a protein shown to be required for correct pheromone detection [50, 66–68]. EbalSNMP2 and EcorSNMP2 are similar to DmelSNMP2, reported to be expressed in supporting cells (Fig. 6) [27, 69, 70].

### Differentially expressed genes (DEGs) analysis

**Fig. 7:**
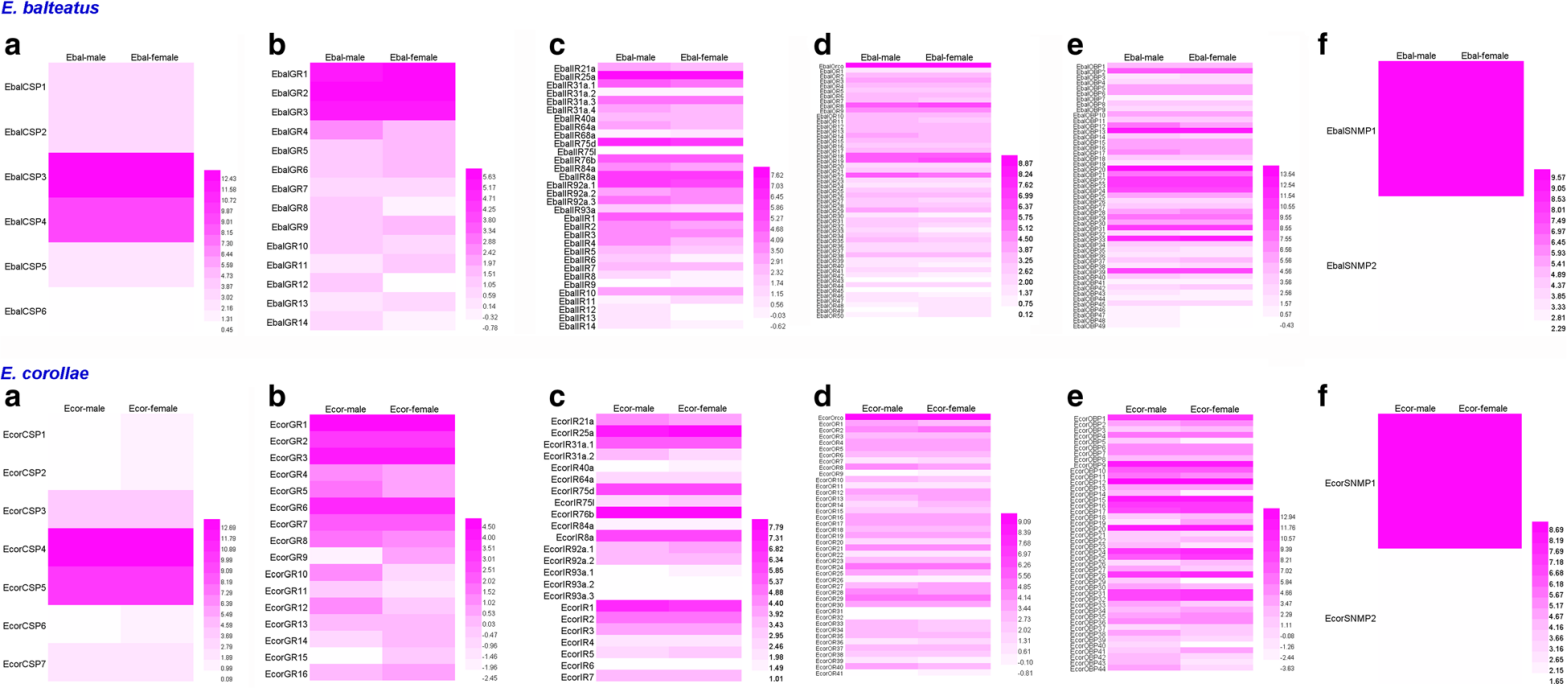
Expression profiles of chemosensory genes in *E. balteatus* and *E. corollae*. **a**: CSPs; **b**: GRs; **c**: IRs; **d**: ORs; **e**: OBPs; and **f**: SNMPs

Gene expression levels of all male and female antennae-associated chemosensory genes in both *E. balteatus* and *E. corollae* were assessed using fragments per kilobase per million fragments (FPKM) values, represented in a heatmap (Fig. 7). Normalised antennal expression levels of candidate *E. balteatus* and *E. corollae* ORs are shown in Additional file 7. Of all ORs, Orco had the highest expression level of transcripts in both sexes of each species. There were no significant differences of OR transcript abundances (FPKM value) in the respective male and female antenna, except for EcorOR14 (Additional file 7). A combined analysis of false discovery rate (FDR) ≤0.001 and |log2 Ratio| ≥ 1 showed that EcorOBP shared highest number of differentially expressed genes (DEGs), including eleven high-expression in male and six high-expression in female syrphids (Additional file 7). In addition, candidate carbon dioxide receptor GR1 and GR2, and SNMP1 in both sexes showed a high expression level (Fig. 7).

### Tissue- and sex- specific expression of candidate *E. balteatus* an*d E. corollae* OR genes

**Fig. 8:**
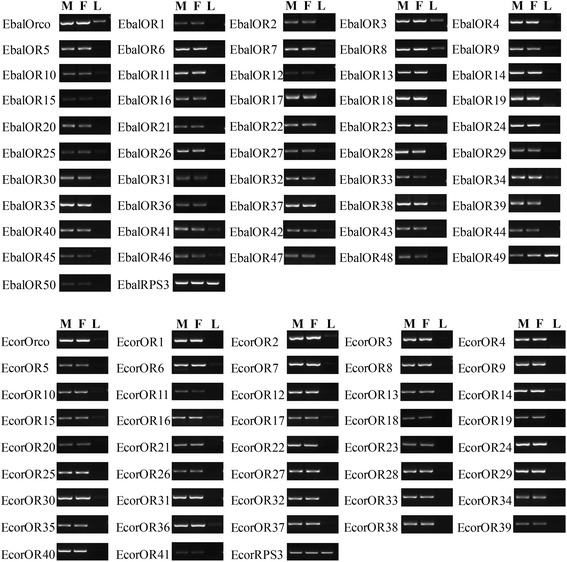
Tissue- and sex- specific expression of candidate *E. balteatus* and *E. corollae* OR genes. M: male antennae, F: female antennae, L: legs (both sexes mixed)

The expression of the candidate ORs in *E. balteatus* an*d E. corollae* male and female antennae and legs (control sample) was analyzed using semi-quantitative reverse transcription PCR (RT-PCR). All 51 EbalORs and 42 EcorORs were detected in the antennae at high expressing level. Only EbalOR49 was found to be mainly expressed in legs. There were no significant differences of transcript abundances in the respective male and female antenna (Fig. 8). The Orco co-receptor gene also showed a high expression level in both syrphid species. This is consistent with DEGs analysis of OR transcript abundances using the FPKM value.

## Discussion

The syrphids *E. balteatus* and *E. corollae* are aphid-specific predators and predominately inhabit northern China wheat and cotton fields. Typical of most insects, chemical cues drive several aspects of their behavior, such as foraging on prey and choosing oviposition sites [7, 8, 10]. Chemosensory proteins play an important role in this process. We analyzed antennal transcriptomes of *E. balteatus* and *E. corollae* and searched for chemosensory genes with the purpose of understanding chemical communication of tritrophic interactions among plants, herbivorous insects, and natural enemies.

In our study, we sequenced *E. balteatus* and *E. corollae* antennal transcriptomes using next generation sequencing technology on the Illumina HiSeq 2000 platform. The total RNA was converted into a template library for high throughput DNA sequencing, allowing us to obtain all expressed transcripts. De novo assembly of transcripts using the Trinity method gives high-efficiency and reliable full-length transcripts across extensive expression levels, even without genome information [71]. Our sequence assembly yielded a final transcript dataset of 50,942 unigenes from *E. corollae* and 53,575 *E. balteatus* unigenes. Total unigenes counts resulted in 44.2% unigenes from *E. balteatus* and 50.3% unigenes from *E. corollae* shared sequence similarities to known proteins using the BLASTX homology search from the NCBI non-redundant protein database. These percentages are very similar to other Dipteran species [50, 72]. Remaining transcripts without associated GO terms may represent species-specific genes. The antennal transcriptome analysis proved to be a powerful tool to identify chemosensory genes in insects without genome information. It has been successfully employed in many insect orders including Lepidoptera, Coleoptera, Hymenoptera and Hemiptera. In Diptera, the chemosensory genes were successfully identified in *C. stygia*, *B. dorsalis* and *Scaeva pyrastri* antennal transcriptomes [50, 72, 73]. Here, we identified 154 and 134 candidate chemosensory genes in *E. balteatus* and *E. corollae*, respectively, a number similar to other Diptera antennal transcriptomes (e.g. 128 in *C. stygia*) [50] but less than the chemosensory genes identified in *D. melanogaster* (254), *M. domestica* (386) and *A. gambiae* (292) genome [30, 33, 47, 49, 63, 65, 74]. This could be the result of differential expression based on developmental stages of the insect larva or adult olfactory organ development such as maxillary palp and proboscis. All data shows that the chemosensory genes identified by antennal transcriptome sequencing are accurate and reliable.

We identified 154 candidate chemosensory genes (51 ORs, 32 IRs, 14 GRs, 49 OBPs, 6 CSPs and 2 SNMPs) in *E. balteatus* and 134 (42 ORs, 23 IRs, 16 GRs, 44 OBPs, 7 CSPs and 2 SNMPs) were identified in *E. corollae*, numbers slightly different compared with those of other Dipteran species [50, 73, 75, 76]. Such differences could be due to sequencing methods, coverage and/or depth. The number of chemosensory genes is higher in *E. balteatus* than in *E. corollae.* However, assembling and splicing quality (unigene number and N50 length) in *E. corollae* is better than in *E. balteatus.* The differences in the number and quality of transcripts identified could arise from variations in sample preparation or could be due to evolution [77] and adaptation to the environment (tritrophic interactions).

A total of 49 and 44 OBPs were identified in *E. balteatus* and *E. corollae* transcriptomes, respectively. The number of OBPs is variable across species, with 52 members in *D. melanogaster*, 66 in *A. gambiae* (Diptera), 21 in *Apis mellifera* (Hymemoptera), 34 in *Helicoverpa armigera*, 29 in *Helicoverpa assulta* (Lepidoptera), 26 in *Colaphellus bowringi*, 46 in *Tribolium castaneum* (Coleoptera) and 15 in *A. pisum* (Hemiptera) [27, 74, 77–83]. Meanwhile, the OBPs of these two syrphid species are highly divergent with those of other insects. These evolutionary differences may result from different physiological functions or ecological niches. Compared with OBPs, only a small amount of CSPs were detected in Diptera. They are only 4 CSPs in *D. melanogaster*, 4 in *C. stygia* and 7 in *A. gambiae* (Diptera). These numbers are much lower than in other insect orders, such as 18 CSPs found in *H. armigera* and 17 in *H. assulta* (Lepidoptera) [27, 50, 80, 81]. In our study, six EbalCSPs and seven EcorCSPs are identified in transcriptome sequencing, revealing that the numbers of CSP gene family differ among species. CSPs show a high evolutionary diversity in insecta, probably related to different physiological functions.

We identified 51 ORs from *E. balteatus* and 42 ORs from *E. corollae*, respectively. Compared with other Dipteran species, these numbers are similar to those identified in *C. stygia* (50) [50] and *G. morsitans morsitans* (46) [51] but lower than those of *D. melanogaster* (62), *M. domestica* (86), *A. gambiae* (79) [47, 49, 84–86], suggesting that sequencing method/depth may be different between studies yielding less genes that may be difficult to detect because of low expression [77]. Here, we were able to detect species-specific OR transcripts in *E. balteatus* and *E. corollae*. This clade of ORs may have a greater impact on recognizing specific odors, particularly perception of aphids-derived volatiles and herbivore-induced plant volatiles granting syrphid localization access of its prey.

The tissue- and sex-specific expression analysis showed no differences between male and female, which is consistent with DEGs analysis of OR transcript abundances using FPKM values. Lepidopteran ORs have shown male-specific expression that is usually involved in the detection of the sex pheromone [19, 26, 27], but this does not seem to be the case in syrphids. Additional real time quantitative PCR, in situ hybridization and single-sensilla recordings would be required to validate OR expressions and functions.

In *D. melanogaster*, males release the volatile sex pheromone *cis*-vaccenyl acetate (cVA) [87–89]. The perception of sex pheromone cVA is mediated by OR67d [88], OR65a [90, 91], LUSH [92], and SNMP1 [34]. In our two syrphid species, EbalOBP17 and EcorOBP14 are the orthologues of the DmelOBP-lush gene, while EbalOR16 and EcorOR24 are the orthologues of DmelOR67d, and EbalSNMP1 and EcorSNMP1 are very similar to DmelSNMP1, suggesting that these proteins may be involved in detection of their yet unidentified pheromones. Therefore, further functional characterization of these candidate proteins will help reveal any mechanism associated with pheromone reception in *E. balteatus* and *E. corollae*.

The *E. balteatus* and *E. corollae* IR family is relatively conserved, especially with respect to common receptors IR8a and IR25a, which are expressed in both olfactory and gustatory systems [30, 45]. The numbers of IRs identified in *E. balteatus* (32) and *E. corollae* (23) are similar to that of *C. stygia* (22) [50], but lower than those of *D. melanogaster* (66) and *A. gambiae* (46) [63]. It is possible that some IRs do not express in antennae tissues or perhaps the number of IRs varies between species and is dependent on natural habitats. A large number of EbalIRs and EcorIRs are clustered with “antennal” orthologues in *Drosophila*, indicating that IRs are highly conserved in Diptera. Furthermore, the IRs identified in these two species may be activated by acids, amines and other odorants that are not sensed by ORs [30, 45, 46].

In the antennae of *E. balteatus* and *E. corollae*, we identified 14 and 16 candidate GRs, respectively. The total number of GRs in these two species may be much larger, because some members could be exclusively expressed in other gustatory organs, such as maxillary palps, proboscises and legs. However, the numbers are still lower than those reported in other Dipteran antennal transcriptomes [50]. The conserved receptors identified in the two syrphid species may be involved in CO_2_ perception. However, we infer that the mechanism of CO_2_ perception is different from mosquitoes which concerns host-seeking [43, 44, 48, 93]. Some GRs may function as taste or contact receptors [31], particularly with reference to their specific pollination behavior [94, 95]. Some GRs from these two species are clustered with thermos-sensing GRs and sugar-detecting GRs from *Drosophila*, indicating that they may perform similar functions. Functional analysis of the candidate *E. balteatus* and *E. corollae* chemosensory proteins is required to identify their physiological roles.

## Conclusions

We have identified and annotated 154 transcripts encoding putative chemosensory proteins in antennal transcriptome of *E. balteatus* and 134 in *E. corollae*. Comparisons between the two syrphid species and among other Dipteran species were deduced using sequence information. This work gives a foundation for future studies aimed at understanding chemical communication in syrphids and tritrophic interactions between plants, herbivorous insects, and natural enemies in agricultural ecosystems.

## Methods

### Insect rearing and tissue collection

*E balteatus* and *E. corollae* larvae were fed with aphids (*Aphis gossypii* Glover) and maintained at 22 ± 1 °C with a 12 h light: 12 h dark photo-period at the Institute of Plant Protection, Chinese Academy of Agricultural Sciences, Beijing, China. Following eclosion, adult males and females were separated and provided with pollen and 10% honey solution.

Antennae were excised from 2- to 5-day-old adult males and females respectively, and legs were collected together, then immediately frozen and stored in liquid nitrogen.

### cDNA library construction and Illumina sequencing

Total RNA of male and female antennae was extracted from *E. balteatus* and *E. corollae* using TRIzol reagent (Invitrogen, Carlsbad, CA, USA). The method for RNA extraction followed in the manufacturer’s instruction. Total RNA was dissolved in RNase-free water and RNA integrity was verified by gel electrophoresis. RNA concentration and purity were measured on a Nanodrop ND-2000 spectrophotometer (NanoDrop products, Wilmington, DE, USA). Ten micrograms total RNA of each sample was used to construct the cDNA library. The cDNA library construction and Illumina HiSeq 2000 (Illumina, San Diego, CA, USA) sequencing of the samples was performed at Beijing Genomics Institute (BGI, Shenzhen, China). The insert sequence length was around 200 bp and these libraries were pair-end sequenced using PE100 strategy [22, 27].

### Assembly and function annotation

Raw reads were pre-processed by filtering low quality reads, trimming low quality nucleotides at each ends and removing 3′ adaptors and poly-A/T tails. Each clean-read dataset of male and female antenna was fed to Trinity [71]. The Trinity assembly procedure, including Inchworm, Chrysalis and Butterfly were followed using Grabherr *et al.*, 2011 as a reference [71]. In the first step of Trinity, Inchworm assembles reads into the unique sequences of transcripts using the default parameters (default *k*-mers = 25). Next, Chrysalis clusters related contigs that correspond to portions of alternatively spliced transcripts or otherwise unique portions of paralogous genes. Finally, Butterfly uses read sequences, read-pairings and Chrysalis’ read mappings to select the paths that are best supported by read sequences [71].

The Trinity outputs were clustered by TGICL [96]. The consensus cluster sequences and singletons make up the unigenes dataset [22]. The unigenes annotation was performed by NCBI BLASTX against a pooled database of non-redundant and SwissProt protein sequences with e-value <1e-5. The BLASTX results were then imported into Blast2GO pipeline for GO annotation [97].

### Identification of chemosensory genes

Candidate unigenes encoding putative ORs, IRs, OBPs, CSPs, SNMPs and GRs were found by running Perl scripts against transcriptome assembly and annotation in the remote sever. Perl scripts were written to extract sequence from functional annotation results using olfaction keywords. Subsequently, all candidate chemosensory genes were manually checked by BLASTX against local non-redundant database with e-value <1e-5. Using the BLASTX NCBI database, we manually performed alignments comparing transcripts against all known proteins to examine full-length coverage. The full-length transcripts contain start and termination codons. The ORFs of all putative chemosensory genes were predicted by using ExPASy (Expert Protein Analysis System) server version (http://web.expasy.org/translate/) according to the BLASTX best hit result [98]. Putative N-terminal signal peptide of OBPs and CSPs were predicted by SignalP 4.0 server version with default parameters [99]. The TMDs of ORs, IRs and GRs were predicted using TMHMM server version 2.0 [100].

### Sequence and phylogenetic analysis

After removing redundancy, alignments of amino acid sequences were performed by MAFFT (https://www.ebi.ac.uk/Tools/msa/mafft/). The phylogenetic trees of *E. balteatus and E. corollae* chemosensory genes were constructed by RaxML version 8 with Jones-Taylor-Thornton amino acid substitution model (JTT) [101] with the putative chemosensory genes in other Dipteran species (Additional file 8: Table S4). Node support was assessed using a bootstrap method based on 1000 replicates. The OR data set contained OR sequences identified in Dipteran (51 from *E balteatus*, 42 from *E. corollae*, 62 from *D. melanogaster* [85, 86], 61 from *B. dorsalis* [genome: assembly ASM78921v2], 50 from *C. stygia* [50] and 81 from *M. domestica* [49]). The GR data set contained GR sequences identified in Dipteran (14 from *E balteatus*, 16 from *E. corollae*, 68 from *D. melanogaster* [47], 40 from *B. dorsalis* [genome: assembly ASM78921v2], 21 from *C. stygia* [50] and 43 from *M. domestica* [49]). The IR data set contained IR sequences identified in Dipteran (32 from *E balteatus*, 23 from *E. corollae*, 76 from *D. melanogaster* [30, 63], 22 from *C. stygia* [50] and 54 from *A. gambiae* [63, 84]). The OBP data set contained OBP sequences identified in Dipteran (49 from *E balteatus*, 44 from *E. corollae*, 71 from *D. melanogaster* [64], 40 from *B. dorsalis* [genome: assembly ASM78921v2], 28 from *C. stygia* [50] and 52 from *M. domestica* [49]). The CSP data set contained CSP sequences identified in Dipteran (7 from *E balteatus*, 9 from *E. corollae*, 4 from *D. melanogaster* [74], 4 from *C. stygia* [50] and 8 from *A. gambiae* [74]). The SNMP data set contained SNMP sequences identified in Dipteran (2 from *E balteatus*, 2 from *E. corollae*, 2 from *D. melanogaster* [34, 35] and 2 from *A. gambiae* [35]).

### DEGs analysis

A mapping-based expression profiling analysis of the chemosensory genes was conducted to compare gene expression between male and female antennae. All of the clean reads were remapped onto the transcripts using SOAPaligner (http://soap.genomics.org.cn /soapaligner.html), allowing up to three base mismatches and a minimum length of 40 bp. The FPKM method was used for calculating unigene expression levels [20, 50, 102, 103]. The suitable *P*-values were calculated to identify differentially expressed genes according to the hypergeometric test [103]. The FDR was a statistical method used in multiple hypothesis testing to correct for *P*-value. Criteria for estimating significant differential expression was set at FDR ≤ 0.001 and |log2 Ratio| ≥ 1. Heatmaps of differential gene expression between male antennae and female antennae in both species were generated by Heml 1.0 software [104].

### Expression analysis by semi-quantitative RT-PCR

Semi-quantitative RT-PCR was performed to verify the expression of candidate chemosensory genes. Male and female antennae and legs were collected from adult *E. balteatus* and *E. corollae* after eclosion. The extraction of total RNA followed the manufacturer’s instruction [27]. The cDNA was synthesized from total RNA using RevertAid First Strand cDNA Synthesis Kit (Thermo Scientific, Waltham, MA, USA). Gene specific primers were designed using PrimerQuest Tool (http://sg.idtdna.com/Primerquest/Home/Index) (Additional file 9: Table S5) and synthesized by Sangon Biotech Co., Ltd. (Shanghai, China). A Taq MasterMix (CWBIO, Beijing, China) was used for PCR reactions under the general three-step amplification of 94 °C for 30s, 55 °C for 30s, 72 °C for 30s. RT-PCR products were separated on 2% agarose gels, stained by ethidium bromide (EB), and photographed under UV light in Gel Doc XR+ Gel Documentation System with Image Lab Software (Bio-Rad, Hercules, CA, USA).

## Additional files

Additional file 1: Table S1. Assembly summary of *E. balteatus* and *E. corollae* antennal transcriptome. (DOCX 16 kb)

Additional file 2: Fig. S1. (A) Species distribution and annotation summaries in the *E. balteatus* (Ebal) and *E. corollae* (Ecor) antennal transcriptome assembly. (B) Gene ontology classifications of the *E. balteatus* and *E. corollae* unigenes with Blast2GO program, including categories with biological process, molecular function and cellular component. (TIFF 3397 kb)

Additional file 3: Table S2. Candidate *E. balteatus* and *E. corollae* antennal chemosensory genes. Unigenes of candidate odorant receptors (2–1), gustatory receptors (2–2), ionotropic receptors (2–3), odorant binding proteins (2–4), chemosensory proteins (2–5) and sensory neuron membrane proteins (2–6) with gene name, length, ORF, best BLASTX hit and identity. (DOCX 112 kb)

Additional file 4: Table S3. Comparison of homologous ORs in *E. balteatus* and *E. corollae*. (DOCX 61 kb)

Additional file 5: Fig. S2. Protein domain analysis of the species-specific IR clade with *Drosophila* iGluRs and DmelIR94d /e. Amino acid alignments shows the ligand binding domains (S1 and S2), the ion channel pore (P), and TMD (M1, M2 and M3) of ionotropic receptors. The key ligand binding residues are marked in red box. (JPEG 4154 kb)

Additional file 6: Fig. S3. Amino acid alignments of the species-specific OBPs clade in the *E. balteatus* and *E. corollae*. The motif of six conserved cysteines are marked with asterisks at the top. (JPEG 3067 kb)

Additional file 7: Antennal expression levels of candidate *E. balteatus* and *E. corollae* odorant receptors. (XLSX 99 kb)

Additional file 8: Table S4. GenBank accession numbers of chemosensory genes used in phylogenetic analyses. (XLSX 34 kb)

Additional file 9: Table S5. Primers of candidate ORs in *E. balteatus* and *E. corollae* used for RT-PCR. (DOCX 20 kb)

## Abbreviations

cDNA: Complementary DNA
CSP: Chemosensory protein
DEGs: Differentially expressed genes
FDR: False discovery rate
FPKM: Fragments per kilobase per million fragments
GLV: Green leaf volatiles
GO: Gene ontology
GR: Gustatory receptor
IR: Ionotropic receptor
OBP: Odorant-binding protein
OR: Odorant receptor
ORF: Open reading frame
PCR: Polymerase chain reaction
RT-PCR: Semi-quantitative reverse transcription PCR
SNMP: Sensory neuron membrane protein
TMD: Transmembrane domain

## References

[1] Amoros-Jimenez R, Robert CA, Marcos-Garcia MA, Fereres A, Turlings TC (2015). A differential role of volatiles from conspecific and heterospecific competitors in the selection of oviposition sites by the Aphidophagous hoverfly *Sphaerophoria rueppellii*. J Chem Ecol.

[2] Brewer MJ, Elliott NC (2004). Biological control of cereal aphids in north america and mediating effects of host plant and habitat manipulations. Annu Rev Entomol.

[3] Amoros-Jimenez R, Pineda A, Fereres A, Marcos-Garcia MA (2012). Prey availability and abiotic requirements of immature stages of the aphid predator *Sphaerophoria rueppellii*. Biol Control.

[4] Freier B, Triltsch H, Mowes M, Moll E (2007). The potential of predators in natural control of aphids in wheat: results of a ten-year field study in two German landscapes. BioControl.

[5] Haenke S, Scheid B, Schaefer M, Tscharntke T, Thies C (2009). Increasing syrphid fly diversity and density in sown flower strips within simple vs. complex landscapes. J Appl Ecol.

[6] Latham DR, Mills NJ (2009). Quantifying insect predation: a comparison of three methods for estimating daily per capita consumption of two Aphidophagous predators. Environ Entomol.

[7] Francis F, Lognay G, Haubruge E (2004). Olfactory responses to aphid and host plant volatile releases: (E)-beta-farnesene an effective kairomone for the predator *Adalia bipunctata*. J Chem Ecol.

[8] Verheggen FJ, Arnaud L, Bartram S, Gohy M, Haubruge E (2008). Aphid and plant volatiles induce oviposition in an aphidophagous hoverfly. J Chem Ecol.

[9] Poppy GM (1997). Tritrophic interactions: improving ecological understanding and biological control?. Endeavour.

[10] Vet LEM, Dicke M (1992). Ecology of infochemical use by natural enemies in a tritrophic context. Annu Rev Entomol.

[11] Bargen H, Saudhof K, Poehling HM (1998). Prey finding by larvae and adult females of *Episyrphus balteatus*. Entomol Exp Appl.

[12] Sadeghi H, Gilbert F (2000). Oviposition preferences of aphidophagous hoverflies. Ecol Entomol.

[13] Sadeghi H, Gilbert F (2000). Aphid suitability and its relationship to oviposition preference in predatory hoverflies. J Anim Ecol.

[14] Zhu J, Obrycki JJ, Ochieng SA, Baker TC, Pickett JA, Smiley D (2005). Attraction of two lacewing species to volatiles produced by host plants and aphid prey. Die Naturwissenschaften.

[15] Almohamad R, Verheggen FJ, Francis F, Haubruge E (2007). Predatory hoverflies select their oviposition site according to aphid host plant and aphid species. Entomol Exp Appl..

[16] Verheggen F, Ryne C, Olsson POC, Arnaud L, Lognay G, Hogberg HE (2007). Electrophysiological and behavioral activity of secondary metabolites in the confused flour beetle. Tribolium confusum J Chem Ecol.

[17] Verheggen FJ, Fagel Q, Heuskin S, Lognay G, Francis F, Haubruge E (2007). Electrophysiological and behavioral responses of the multicolored asian lady beetle, *Harmonia axyridis* Pallas, to sesquiterpene semiochemicals. J Chem Ecol.

[18] Suh E, Bohbot J, Zwiebel LJ (2014). Peripheral olfactory signaling in insects. Curr opin insect sci.

[19] Liu Y, Gu SH, Zhang YJ, Guo YY, Wang GR (2012). Candidate olfaction genes identified within the *Helicoverpa armigera* antennal transcriptome. PLoS One.

[20] Andersson MN, Videvall E, Walden KKO, Harris MO, Robertson HM, Lofstedt C (2014). Sex- and tissue-specific profiles of chemosensory gene expression in a herbivorous gall-inducing fly (Diptera: Cecidomyiidae). BMC Genomics.

[21] Cao DP, Liu Y, Walker WB, Li JH, Wang GR (2014). Molecular characterization of the *Aphis gossypii* olfactory receptor gene families. PLoS One.

[22] Cao DP, Liu Y, Wei JJ, Liao XY, Walker WB, Li JH (2014). Identification of candidate olfactory genes in *Chilo suppressalis* by antennal transcriptome analysis. Int J Biol Sci.

[23] Dippel S, Oberhofer G, Kahnt J, Gerischer L, Opitz L, Schachtner J (2014). Tissue-specific transcriptomics, chromosomal localization, and phylogeny of chemosensory and odorant binding proteins from the red flour beetle *Tribolium castaneum* reveal subgroup specificities for olfaction or more general functions. BMC Genomics.

[24] Gu SH, Sun L, Yang RN, Wu KM, Guo YY, Li XC (2014). Molecular characterization and differential expression of olfactory genes in the antennae of the black cutworm moth *Agrotis ipsilon*. PLoS One.

[25] Hodges TK, Cosme LV, Athrey G, Pathikonda S, Takken W, Slotman MA. Species-specific chemosensory gene expression in the olfactory organs of the malaria vector *Anopheles gambiae* (Retracted article. See vol. 16, 572, 2015). BMC genomics*.* 2014; 15:1089. 10.1186/1471-2164-15-1089 PMC4299676 25495232

[26] Zhang SF, Zhang Z, Wang HB, Kong XB (2014). Antennal transcriptome analysis and comparison of olfactory genes in two sympatric defoliators, *Dendrolimus houi* and *Dendrolimus kikuchii* (Lepidoptera: Lasiocampidae). Insect Biochem Mol Biol.

[27] Zhang J, Wang B, Dong SL, Cao DP, Dong JF, Walker WB (2015). Antennal transcriptome analysis and comparison of chemosensory gene families in two closely related Noctuidae moths, *Helicoverpa armigera* and *H. assulta*. PloS one.

[28] Yang B, Ozaki K, Ishikawa Y, Matsuo T (2015). Identification of candidate odorant receptors in Asian corn borer *Ostrinia furnacalis*. PLoS One.

[29] Buck L, Axel R (1991). A novel multigene family may encode odorant receptors: a molecular basis for odor recognition. Cell.

[30] Benton R, Vannice KS, Gomez-Diaz C, Vosshall LB (2009). Variant ionotropic glutamate receptors as chemosensory receptors in *Drosophila*. Cell.

[31] Clyne PJ, Warr CG, Carlson JR (2000). Candidate taste receptors in *Drosophila*. Sci.

[32] Pelosi P, Zhou JJ, Ban LP, Calvello M (2006). Soluble proteins in insect chemical communication. Cell Mol Life Sci.

[33] Pelosi P, Iovinella I, Felicioli A, Dani FR (2014). Soluble proteins of chemical communication: an overview across arthropods. Front Physiol.

[34] Benton R, Vannice KS, Vosshall LB (2007). An essential role for a CD36-related receptor in pheromone detection in *Drosophila*. Nat.

[35] Vogt RG, Miller NE, Litvack R, Fandino RA, Sparks J, Staples J (2009). The insect SNMP gene family. Insect Biochem Mol Biol.

[36] Jin X, Ha TS, Smith DP (2008). SNMP is a signaling component required for pheromone sensitivity in *Drosophila*. Proc Natl Acad Sci U S A.

[37] Song HG, Kwon JY, Han HS, Bae YC, Moon C (2008). First contact to odors: our current knowledge about odorant receptors. Sensors.

[38] Kirkness EF, Haas BJ, Sun WL, Braig HR, Perotti MA, Clark JM (2010). Genome sequences of the human body louse and its primary endosymbiont provide insights into the permanent parasitic lifestyle. Proc Natl Acad Sci U S A.

[39] Zhou XF, Slone JD, Rokas A, Berger SL, Liebig J, Ray A (2012). Phylogenetic and transcriptomic analysis of chemosensory receptors in a pair of divergent ant species reveals sex-specific signatures of odor coding. PLoS Genet.

[40] Hallem EA, Ho MG, Carlson JR (2004). The molecular basis of odor coding in the *Drosophila* antenna. Cell.

[41] Hallem EA, Carlson JR (2006). Coding of odors by a receptor repertoire. Cell.

[42] Kwon JY, Dahanukar A, Weiss LA, Carlson JR (2007). The molecular basis of CO2 reception in *Drosophila*. Proc Natl Acad Sci U S A.

[43] Gillies MT (1980). The role of carbon dioxide in host-finding by mosquitoes (Diptera: Culicidae): a review. Bull Entomol Res.

[44] Erdelyan CNG, Mahood TH, Bader TSY, Whyard S (2012). Functional validation of the carbon dioxide receptor genes in *Aedes aegypti* mosquitoes using RNA interference. Insect Mol Biol.

[45] Ai MR, Blais S, Park JY, Min S, Neubert TA, Suh GSB (2013). Ionotropic glutamate receptors IR64a and IR8a form a functional odorant receptor complex *in vivo* in *Drosophila*. J Neurosci.

[46] Ai M, Min S, Grosjean Y, Leblanc C, Bell R, Benton R (2010). Acid sensing by the *Drosophila* olfactory system. Nat.

[47] Robertson HM, Warr CG, Carlson JR (2003). Molecular evolution of the insect chemoreceptor gene superfamily in *Drosophila melanogaster*. Proc Natl Acad Sci U S A.

[48] Rinker DC, Zhou XF, Pitts RJ, Rokas A, Zwiebel LJ, Consortium A (2013). Antennal transcriptome profiles of anopheline mosquitoes reveal human host olfactory specialization in *Anopheles gambiae*. BMC Genomics.

[49] Scott JG, Warren WC, Beukeboom LW, Bopp D, Clark AG, Giers SD (2014). Genome of the house fly, *Musca domestica* L., a global vector of diseases with adaptations to a septic environment. Genome Biol.

[50] Leitch O, Papanicolaou A, Lennard C, Kirkbride KP, Anderson A (2015). Chemosensory genes identified in the antennal transcriptome of the blowfly *Calliphora stygia*. BMC Genomics.

[51] Obiero GF, Mireji PO, Nyanjom SR, Christoffels A, Robertson HM, Masiga DK (2014). Odorant and gustatory receptors in the tsetse fly *Glossina morsitans morsitans*. PLoS Negl Trop Dis.

[52] Francis FD, Martin T, Lognay G, Haubruge E (2005). Role of (E)-beta-farnesene in systematic aphid prey location by *Episyrphus balteatus* larvae (Diptera : Syrphidae). Eur J Entomol.

[53] Harmel N, Almohamad R, Fauconnier ML, Du Jardin P, Verheggen F, Marlier M (2007). Role of terpenes from aphid-infested potato on searching and oviposition behavior of *Episyrphus balteatus*. Insect Sci.

[54] Turlings TCJ, Tumlinson JH (1992). Systemic release of chemical signals by herbivore-injured corn. Proc Natl Acad Sci U S A.

[55] Pare PW, Tumlinson JH (1997). De novo biosynthesis of volatiles induced by insect herbivory in cotton plants. Plant Physiol.

[56] Jones WD, Cayirlioglu P, Kadow IG, Vosshall LB (2007). Two chemosensory receptors together mediate carbon dioxide detection in *Drosophila*. Nat.

[57] Ni L, Bronk P, Chang EC, Lowell AM, Flam JO, Panzano VC (2013). A gustatory receptor paralogue controls rapid warmth avoidance in *Drosophila*. Nat.

[58] Dahanukar A, Lei YT, Kwon JY, Carlson JR (2007). Two gr genes underlie sugar reception in *Drosophila*. Neuron.

[59] Slone J, Daniels J, Amrein H (2007). Sugar receptors in *Drosophila*. Curr Biol.

[60] Jiao Y, Moon SJ, Montell C (2007). A *Drosophila* gustatory receptor required for the responses to sucrose, glucose, and maltose identified by mRNA tagging. Proc Natl Acad Sci U S A.

[61] Jiao Y, Moon SJ, Wang X, Ren Q, Montell C (2008). Gr64f is required in combination with other gustatory receptors for sugar detection in *Drosophila*. Curr Biol.

[62] Fujii S, Yavuz A, Slone J, Jagge C, Song X, Amrein H (2015). *Drosophila* sugar receptors in sweet taste perception, olfaction, and internal nutrient sensing. Curr Biol.

[63] Croset V, Rytz R, Cummins SF, Budd A, Brawand D, Kaessmann H (2010). Ancient protostome origin of chemosensory ionotropic glutamate receptors and the evolution of insect taste and olfaction. PLoS Genet.

[64] Hekmat-Scafe D, Scafe CR, McKinney AJ, Tanouye MA (2002). Genome-wide analysis of the odorant-binding protein gene familiy in *Drosophila melanogaster*. Genome Res.

[65] Sanchez-Gracia A, Vieira FG, Rozas J (2009). Molecular evolution of the major chemosensory gene families in insects. Heredity.

[66] Krieger J, Raming K, Dewer YME, Bette S, Conzelmann S, Breer H (2002). A divergent gene family encoding candidate olfactory receptors of the moth *Heliothis virescens*. Eur J Neurosci.

[67] Rogers ME, Sun M, Lerner MR, Vogt RG (1997). Snmp-1, a novel membrane protein of olfactory neurons of the silk moth *Antheraea polyphemus* with homology to the CD36 family of membrane proteins. J Biol Chem.

[68] Rogers ME, Krieger J, Vogt RG (2001). Antennal SNMPs (sensor neuron membrane proteins) of lepidoptera define a unique family of invertebrate CD36-like proteins. J Neurobiol.

[69] Forstner M, Gohl T, Gondesen I, Raming K, Breer H, Krieger J (2008). Differential expression of SNMP-1 and SNMP-2 proteins in pheromone-sensitive hairs of moths. Chem Senses.

[70] Gu SH, Yang RN, Guo MB, Wang GR, Wu KM, Guo YY (2013). Molecular identification and differential expression of sensory neuron membrane proteins in the antennae of the black cutworm moth *Agrotis ipsilon*. J Insect Physiol.

[71] Grabherr MG, Haas BJ, Yassour M, Levin JZ, Thompson DA, Amit I (2011). Full-length transcriptome assembly from RNA-Seq data without a reference genome. Nat Biotechnol.

[72] Li XM, Zhu XY, He P, Xu L, Sun L, Chen L (2016). Molecular characterization and sex distribution of chemosensory receptor gene family based on transcriptome analysis of *Scaeva pyrastri*. PLoS One.

[73] Liu Z, Smagghe G, Lei Z, Wang JJ (2016). Identification of male- and female-specific olfaction genes in antennae of the oriental fruit fly (*Bactrocera dorsalis*). PLoS One.

[74] Vieira FG, Rozas J (2011). Comparative genomics of the odorant-binding and chemosensory protein gene families across the Arthropoda: origin and evolutionary history of the chemosensory system. Genome Biol Evol.

[75] Pitts RJ, Rinker DC, Jones PL, Rokas A, Zwiebel LJ (2011). Transcriptome profiling of chemosensory appendages in the malaria vector *Anopheles gambiae* reveals tissue- and sex-specific signatures of odor coding. BMC Genomics.

[76] Riveron J, Boto T, Alcorta E (2013). Transcriptional basis of the acclimation to high environmental temperature at the olfactory receptor organs of *Drosophila melanogaster*. BMC Genomics.

[77] Li XM, Zhu XY, Wang ZQ, Wang Y, He P, Chen G (2015). Candidate chemosensory genes identified in *Colaphellus bowringi* by antennal transcriptome analysis. BMC Genomics.

[78] Foret S, Maleszka R (2006). Function and evolution of a gene family encoding odorant binding-like proteins in a social insect, the honey bee (*Apis mellifera*). Genome Res.

[79] Foret S, Wanner KW, Maleszka R (2007). Chemosensory proteins in the honey bee: insights from the annotated genome, comparative analyses and expressional profiling. Insect Biochem Mol Biol.

[80] Wanner KW, Willis LG, Theilmann DA, Isman MB, Feng QL, Plettner E (2004). Analysis of the insect os-d-like gene family. J Chem Ecol.

[81] Zhou JJ, Vieira FG, He XL, Smadja C, Liu R, Rozas J, Field LM (2010). Genome annotation and comparative analyses of the odorant-binding proteins and chemosensory proteins in the pea aphid *Acyrthosiphon pisum*. Insect Mol Biol.

[82] Wang LJ, Wang SZ, Li YH, Paradesi MSR, Brown SJ (2007). BeetleBase: the model organism database for *Tribolium castaneum*. Nucleic Acids Res.

[83] Xu PX, Zwiebel LJ, Smith DP (2003). Identification of a distinct family of genes encoding atypical odorant-binding proteins in the malaria vector mosquito. Anopheles gambiae Insect Mol Biol.

[84] Fox AN, Pitts RJ, Robertson HM, Carlson JR, Zwiebel LJ (2001). Candidate odorant receptors from the malaria vector mosquito *Anopheles gambiae* and evidence of down-regulation in response to blood feeding. Proc Natl Acad Sci U S A.

[85] Clyne PJ, Warr CG, Freeman MR, Lessing D, Kim JH, Carlson JR (1999). A novel family of divergent seven-transmembrane proteins: candidate odorant receptors in *Drosophila*. Neuron.

[86] Gao Q, Chess A (1999). Identification of candidate *Drosophila* olfactory receptors from genomic DNA sequence. Genomics.

[87] Bartelt RJ, Schaner AM, Jackson LL (1985). Cis-Vaccenyl acetate as an aggregation pheromone in *Drosophila melanogaster*. J Chem Ecol.

[88] Kurtovic A, Widmer A, Dickson BJ (2007). A single class of olfactory neurons mediates behavioural responses to a *Drosophila* sex pheromone. Nat.

[89] Lebreton S, Trona F, Borrero-Echeverry F, Bilz F, Grabe V, Becher PG (2015). Feeding regulates sex pheromone attraction and courtship in *Drosophila* females. Sci Rep.

[90] Liu W, Liang X, Gong J, Yang Z, Zhang YH, Zhang JX (2011). Social regulation of aggression by pheromonal activation of Or65a olfactory neurons in *Drosophila*. Nat Neurosci.

[91] Lebreton S, Grabe V, Omondi AB, Ignell R, Becher PG, Hansson BS (2014). Love makes smell blind: mating suppresses pheromone attraction in *Drosophila* females via Or65a olfactory neurons. Sci Rep.

[92] Kim MS, Repp A, Smith DP (1998). LUSH odorant-binding protein mediates chemosensory responses to alcohols in *Drosophila melanogaster*. Genetics.

[93] Dekker T, Geier M, Carde RT (2005). Carbon dioxide instantly sensitizes female yellow fever mosquitoes to human skin odours. J Exp Biol.

[94] Jauker F, Diekotter T, Schwarzbach F, Wolters V (2009). Pollinator dispersal in an agricultural matrix: opposing responses of wild bees and hoverflies to landscape structure and distance from main habitat. Landsc Ecol.

[95] Raymond L, Plantegenest M, Vialatte A (2013). Migration and dispersal may drive to high genetic variation and significant genetic mixing: the case of two agriculturally important, continental hoverflies (*Episyrphus balteatus* and *Sphaerophoria scripta*). Mol Ecol.

[96] Pertea G, Huang XQ, Liang F, Antonescu V, Sultana R, Karamycheva S (2003). TIGR gene indices clustering tools (TGICL): a software system for fast clustering of large EST datasets. Bioinformatics.

[97] Conesa A, Gotz S, Garcia-Gomez JM, Terol J, Talon M, Robles M (2005). Blast2GO: a universal tool for annotation, visualization and analysis in functional genomics research. Bioinformatics.

[98] Gasteiger E, Gattiker A, Hoogland C, Ivanyi I, Appel RD, Bairoch A (2003). ExPASy: the proteomics server for in-depth protein knowledge and analysis. Nucleic Acids Res.

[99] Petersen TN, Brunak S, von Heijne G, Nielsen H (2011). SignalP 4.0: discriminating signal peptides from transmembrane regions. Nat Methods.

[100] Krogh A, Larsson B, von Heijne G, Sonnhammer ELL (2001). Predicting transmembrane protein topology with a hidden Markov model: application to complete genomes. J Mol Biol.

[101] Stamatakis A (2014). RAxML version 8: a tool for phylogenetic analysis and post-analysis of large phylogenies. Bioinformatics.

[102] Mortazavi A, Williams BA, McCue K, Schaeffer L, Wold B (2008). Mapping and quantifying mammalian transcriptomes by RNA-Seq. Nat Methods.

[103] Audic S, Claverie JM (1997). The significance of digital gene expression profiles. Genome Res.

[104] Deng WK, Wang YB, Liu ZX, Cheng H, Xue Y (2014). HemI: a toolkit for illustrating Heatmaps. PLoS One.

