# Supplementary material for: Chemosensory genes in the antennal transcriptome of two syrphid species,Episyrphus balteatusandEupeodes corollae (Diptera: Syrphidae)
Source: BMC Genomics. 2017 Aug 7;18:586. doi: 10.1186/s12864-017-3939-4 (PMC5547493; doi:10.1186/s12864-017-3939-4)
Supplement: Supplementary file 1 — Assembly summary of E. balteatus and E. corollae antennal transcriptome. (DOCX 16 kb) [file 12864_2017_3939_MOESM1_ESM.docx]

Table S1. Assembly summary of *E. balteatus* and *E. corollae* antennal transcriptome.

|  | Sample | Total Number | Total Length(nt) | Mean Length (nt) | N50  (nt) | Total Consensus Sequences | Distinct Clusters | Distinct Singletons |
| --- | --- | --- | --- | --- | --- | --- | --- | --- |
| ***E. balteatus*** | | | | | | | | |
| Contig | Male | 116,907 | 37,284,464 | 319 | 630 | - | - | - |
|  | Female | 140,267 | 42,441,506 | 303 | 537 | - | - | - |
| Unigene | Male | 57,950 | 39,663,135 | 684 | 1532 | 57,950 | 14,528 | 43,422 |
|  | Female | 68,165 | 44,954,074 | 659 | 1465 | 68,165 | 16,792 | 51,373 |
|  | All | 53,575 | 47,606,367 | 889 | 1724 | 53,575 | 17,407 | 36,168 |
| ***E. corollae*** | | | | | | | | |
| Contig | Male | 104856 | 39245574 | 374 | 1011 | - | - | - |
|  | Female | 128384 | 41953978 | 327 | 730 | - | - | - |
| Unigene | Male | 54116 | 46063286 | 851 | 2021 | 54116 | 15012 | 39104 |
|  | Female | 61220 | 45820937 | 748 | 1757 | 61220 | 18755 | 42465 |
|  | All | 50942 | 52947324 | 1039 | 2104 | 50942 | 18054 | 32888 |
