# Supplementary figures and images for: Chemosensory genes in the antennal transcriptome of two syrphid species,Episyrphus balteatusandEupeodes corollae (Diptera: Syrphidae)
Source: BMC Genomics. 2017 Aug 7;18:586. doi: 10.1186/s12864-017-3939-4 (PMC5547493; doi:10.1186/s12864-017-3939-4)

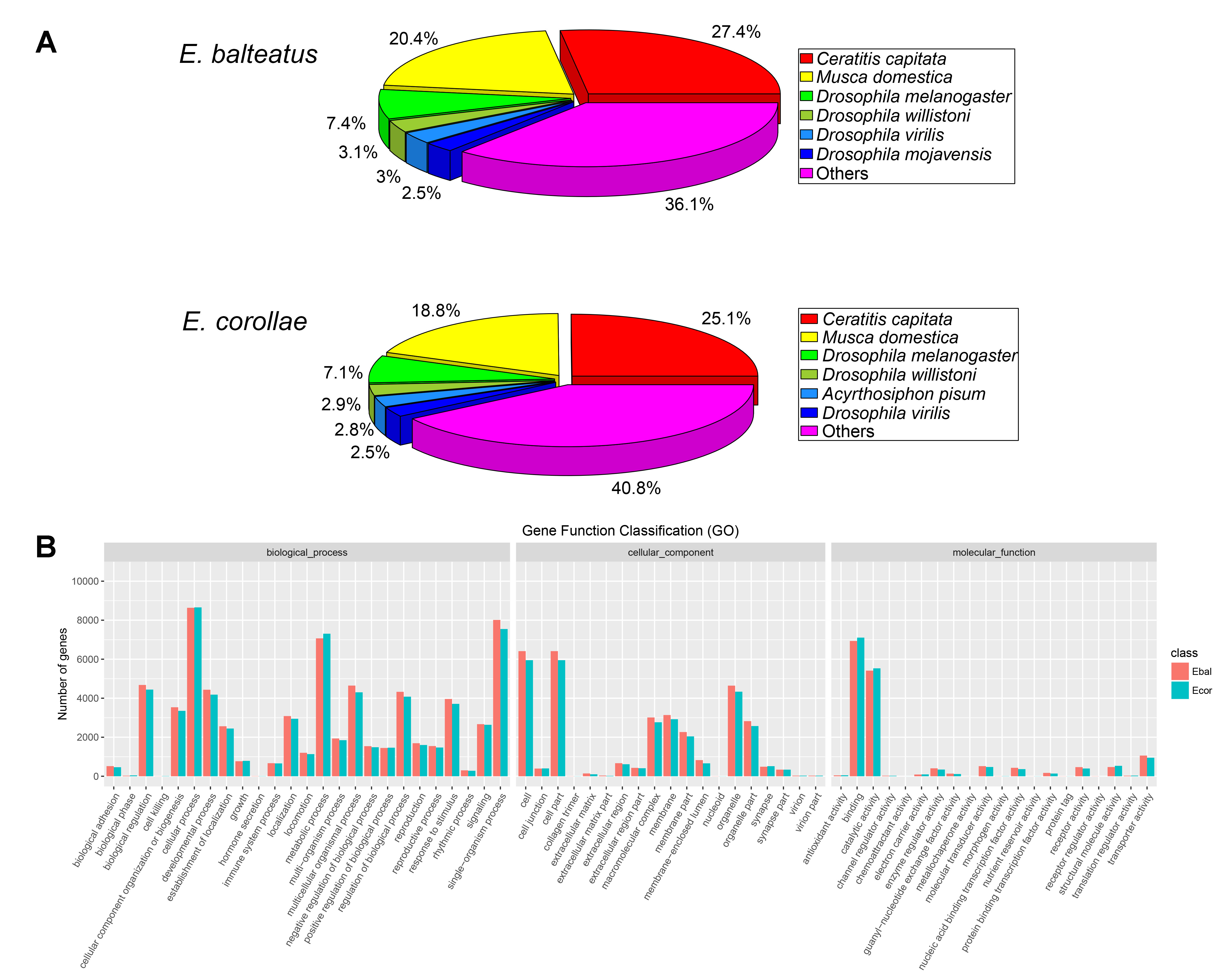

Supplement: Supplementary file 2 — (A) Species distribution and annotation summaries in the E. balteatus (Ebal) and E. corollae (Ecor) antennal transcriptome assembly. (B) Gene ontology classifications of the E. balteatus and E. corollae unigenes with Blast2GO program, including categories with biological process, molecular function and cellular component. (TIFF 3397 kb) [file 12864_2017_3939_MOESM2_ESM.tif]

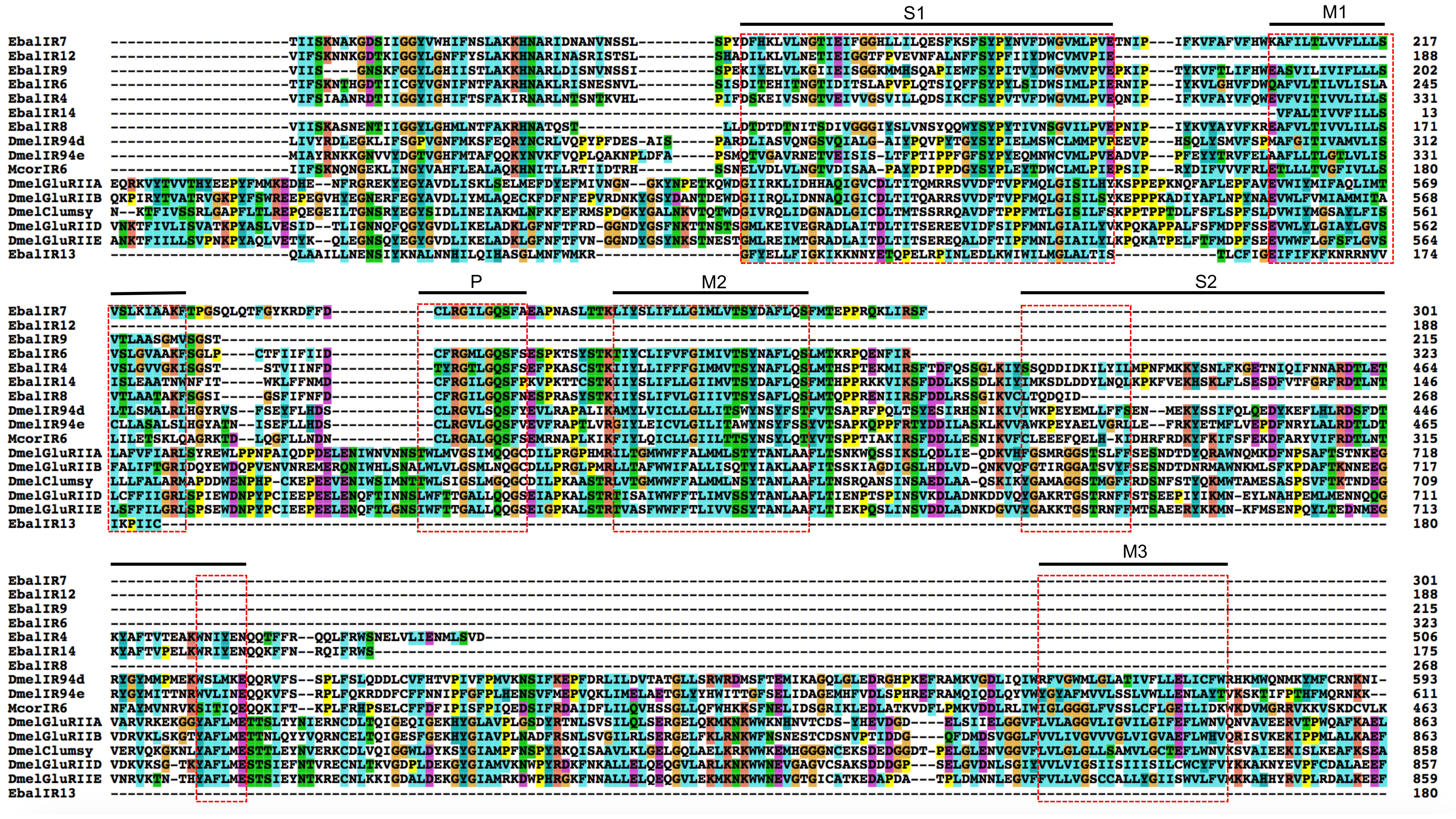

Supplement: Supplementary file 5 — Protein domain analysis of the species-specific IR clade with Drosophila iGluRs and DmelIR94d /e. Amino acid alignments shows the ligand binding domains (S1 and S2), the ion channel pore (P), and TMD (M1, M2 and M3) of ionotropic receptors. The key ligand binding residues are marked in red box. (JPEG 4154 kb) [file 12864_2017_3939_MOESM5_ESM.jpg]

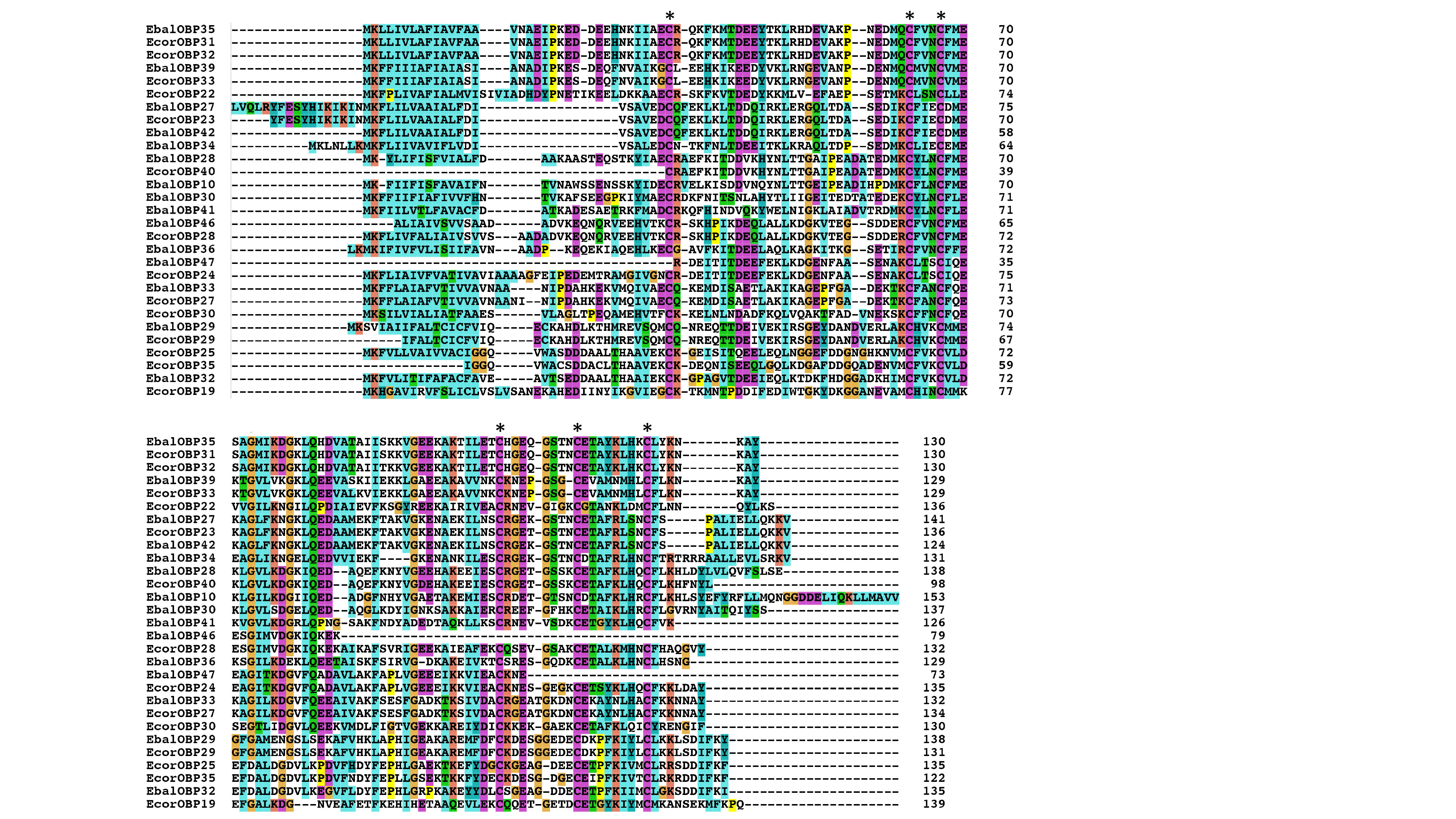

Supplement: Supplementary file 6 — Amino acid alignments of the species-specific OBPs clade in the E. balteatus and E. corollae. The motif of six conserved cysteines are marked with asterisks at the top. (JPEG 3067 kb) [file 12864_2017_3939_MOESM6_ESM.jpg]
