# Supplementary material for: Chemosensory genes in the antennal transcriptome of two syrphid species,Episyrphus balteatusandEupeodes corollae (Diptera: Syrphidae)
Source: BMC Genomics. 2017 Aug 7;18:586. doi: 10.1186/s12864-017-3939-4 (PMC5547493; doi:10.1186/s12864-017-3939-4)
Supplement: Supplementary file 3 — Candidate E. balteatus and E. corollae antennal chemosensory genes. Unigenes of candidate odorant receptors (2–1), gustatory receptors (2–2), ionotropic receptors (2–3), odorant binding proteins (2–4), chemosensory proteins (2–5) and sensory neuron membrane proteins (2–6) with gene name, length, ORF, best BLASTX hit and identity. (DOCX 112 kb) [file 12864_2017_3939_MOESM3_ESM.docx]

Table S2-1. Unigenes of candidate odorant receptors in *E. balteatus* and *E. corollae*

| Unigene reference | Gene name | Length (nt) | ORF (aa) | Blastx best hit (Reference/Name/Species) | Evalue | Identity | TMD (No.) | Full length |
| --- | --- | --- | --- | --- | --- | --- | --- | --- |
| ***E. balteatus*** |  |  |  |  |  |  |  |  |
| Unigene13965 | EbalOrco | 2193 | 476 | gb\|AEA30004.2\| odorant receptor co-receptor [Chrysomya megacephala] | 0 | 86% | 7 | Yes |
| Unigene15361 | EbalOR1 | 1324 | 435 | ref\|XP_011212431.1\| PREDICTED: odorant receptor 49a-like [Bactrocera dorsalis] | 3.00E-58 | 31% | 5 | Yes |
| Unigene5165 | EbalOR2 | 1321 | 425 | ref\|XP_011177369.1\| PREDICTED: odorant receptor 13a [Bactrocera cucurbitae] | 1.00E-171 | 56% | 5 | Yes |
| Unigene9434 | EbalOR3 | 1573 | 421 | gb\|AKI29034.1\| odorant receptor 35a [Bactrocera dorsalis] | 1.00E-87 | 36% | 7 | Yes |
| Unigene11610 | EbalOR4 | 1273 | 419 | gb\|AID61213.1\| odorant receptor, partial [Calliphora stygia] | 1.00E-98 | 42% | 6 | No |
| Unigene1522 | EbalOR5 | 1443 | 413 | gb\|AKI29034.1\| odorant receptor 35a [Bactrocera dorsalis] | 2.00E-66 | 31% | 6 | Yes |
| Unigene6184 | EbalOR6 | 1453 | 406 | gb\|AID61234.1\| odorant receptor, partial [Calliphora stygia] | 6.00E-09 | 25% | 7 | No |
| Unigene21130 | EbalOR7 | 1319 | 402 | ref\|XP_011210110.1\| PREDICTED: odorant receptor 22c [Bactrocera dorsalis] | 4.00E-53 | 28% | 3 | Yes |
| Unigene22458 | EbalOR8 | 1390 | 402 | ref\|XP_011300023.1\| PREDICTED: odorant receptor 13a-like [Fopius arisanus] | 1.00E-76 | 32% | 4 | Yes |
| Unigene10783 | EbalOR9 | 1429 | 400 | gb\|AKI29045.1\| odorant receptor 67d [Bactrocera dorsalis] | 1.00E-106 | 40% | 6 | No |
| Unigene17964 | EbalOR10 | 1247 | 400 | ref\|XP_011210110.1\| PREDICTED: odorant receptor 22c [Bactrocera dorsalis] | 4.00E-61 | 31% | 4 | Yes |
| Unigene18693 | EbalOR11 | 1236 | 400 | ref\|XP_005180133.1\| PREDICTED: putative odorant receptor 69a, isoform B [Musca domestica] | 1.00E-130 | 50% | 6 | Yes |
| Unigene1710 | EbalOR12 | 1200 | 399 | ref\|XP_011179733.1\|odorant receptor 94a-like [Bactrocera cucurbitae] | 2.00E-116 | 45% | 6 | Yes |
| Unigene17686 | EbalOR13 | 1354 | 398 | ref\|XP_011210110.1\| PREDICTED: odorant receptor 22c [Bactrocera dorsalis] | 1.00E-128 | 46% | 6 | Yes |
| CL5748.Contig1 | EbalOR14 | 1545 | 397 | ref\|XP_011200401.1\| PREDICTED: odorant receptor 67c-like [Bactrocera dorsalis] | 1.00E-89 | 38% | 4 | Yes |
| Unigene11857 | EbalOR15 | 1268 | 395 | ref\|XP_004531172.1\| PREDICTED: odorant receptor 7a-like [Ceratitis capitata] | 6.00E-89 | 37% | 6 | Yes |
| Unigene1578 | EbalOR16 | 1182 | 393 | [gb\|KNC23896.1\|putative odorant receptor 67d [Lucilia cuprina]](http://www.ncbi.nlm.nih.gov/protein/906461394?report=genbank&log$=protalign&blast_rank=1&RID=8U4ZRPUM01R) | 9.00E-121 | 43% | 6 | Yes |
| Unigene4088 | EbalOR17 | 1217 | 392 | gb\|AID61212.1\| odorant receptor, partial [Calliphora stygia] | 6.00E-99 | 40% | 6 | No |
| Unigene13298 | EbalOR18 | 1190 | 387 | ref\|XP_011203703.1\| PREDICTED: odorant receptor 67d-like [Bactrocera dorsalis] | 1.00E-108 | 42% | 6 | No |
| Unigene8750ll | EbalOR19 | 1208 | 386 | gb\|AID61215.1\| odorant receptor [Calliphora stygia] | 1.00E-120 | 47% | 6 | Yes |
| Unigene4097 | EbalOR20 | 1161 | 386 | gb\|AID61224.1\|odorant receptor [Calliphora stygia] | 2.00E-137 | 53% | 6 | Yes |
| CL41.Contig1 | EbalOR21 | 1210 | 385 | ref\|XP_011180402.1\| PREDICTED: odorant receptor 94a-like [Bactrocera cucurbitae] | 1.00E-79 | 35% | 5 | Yes |
| Unigene6078 | EbalOR22 | 1270 | 384 | ref\|XP_011203703.1\| PREDICTED: odorant receptor 67d-like [Bactrocera dorsalis] | 1.00E-110 | 42% | 6 | Yes |
| Unigene21072 | EbalOR23 | 1217 | 383 | gb\|AID61232.1\| odorant receptor [Calliphora stygia] | 1.00E-160 | 60% | 5 | No |
| CL1522.Contig2 | EbalOR24 | 1246 | 383 | ref\|XP_011208819.1\| PREDICTED: putative odorant receptor 92a [Bactrocera dorsalis] | 5.00E-57 | 33% | 6 | Yes |
| Unigene6351 | EbalOR25 | 1201 | 382 | gb\|AID61202.1\| odorant receptor [Calliphora stygia] | 1.00E-137 | 53% | 5 | Yes |
| CL6664.Contig1 | EbalOR26 | 1176 | 381 | ref\|XP_008560863.1\| PREDICTED: odorant receptor 83a-like [Microplitis demolitor] | 2.00E-17 | 22% | 6 | No |
| CL1522.Contig3 | EbalOR27 | 1234 | 381 | ref\|XP_011208819.1\| PREDICTED: putative odorant receptor 92a [Bactrocera dorsalis] | 6.00E-71 | 32% | 5 | No |
| Unigene21149 | EbalOR28 | 1134 | 377 | gb\|KNC33320.1\|putative odorant receptor 7a [Lucilia cuprina] | 8.00E-82 | 38% | 4 | No |
| Unigene7167 | EbalOR29 | 1180 | 371 | gb\|AID61215.1\| odorant receptor [Calliphora stygia] | 1.00E-179 | 66% | 4 | Yes |
| CL1028.Contig3 | EbalOR30 | 1095 | 364 | ref\|XP_005178484.1\| PREDICTED: odorant receptor 10a [Musca domestica] | 1.00E-29 | 26% | 5 | No |
| Unigene4038 | EbalOR31 | 1133 | 362 | gb\|EEZ99229.1\| odorant receptor 37 [Tribolium castaneum] | 6.00E-19 | 23% | 6 | No |
| Unigene15652 | EbalOR32 | 1062 | 353 | ref\|XP_005178484.1\| PREDICTED: odorant receptor 10a [Musca domestica] | 1.00E-32 | 26% | 6 | No |
| Unigene16930 | EbalOR33 | 1038 | 345 | gb\|KNC29284.1\|Odorant receptor 43a [Lucilia cuprina] | 2.00E-147 | 59% | 6 | No |
| Unigene2481 | EbalOR34 | 1009 | 312 | gb\|AID61211.1\| odorant receptor [Calliphora stygia] | 8.00E-85 | 44% | 4 | No |
| Unigene4769 | EbalOR35 | 914 | 274 | ref\|XP_011208819.1\| PREDICTED: putative odorant receptor 92a [Bactrocera dorsalis] | 9.00E-55 | 34% | 3 | No |
| Unigene4789 | EbalOR36 | 819 | 261 | ref\|XP_011184142.1\| PREDICTED: odorant receptor 83a-like [Bactrocera cucurbitae] | 9.00E-24 | 32% | 4 | No |
| CL1491.Contig2 | EbalOR37 | 843 | 259 | gb\|AID61221.1\| odorant receptor, partial [Calliphora stygia] | 9.00E-78 | 46% | 4 | No |
| Unigene28390 | EbalOR38 | 785 | 254 | ref\|XP_011208819.1\| PREDICTED: putative odorant receptor 92a [Bactrocera dorsalis] | 8.00E-25 | 29% | 4 | No |
| Unigene18618 | EbalOR39 | 740 | 236 | ref\|XP_005178182.1\| PREDICTED: odorant receptor 63a [Musca domestica] | 3.00E-86 | 52% | 3 | No |
| Unigene5015 | EbalOR40 | 693 | 230 | ref\|XP_012159289.1\| PREDICTED: putative odorant receptor 69a, isoform B [Ceratitis capitata] | 2.00E-21 | 28% | 3 | No |
| CL1028.Contig2 | EbalOR41 | 755 | 230 | ref\|XP_001356952.1\|gb\|EAL34018.1\| Or22c [Drosophila pseudoobscura pseudoobscura] | 2.00E-36 | 33% | 2 | No |
| Unigene2279 | EbalOR42 | 687 | 228 | ref\|XP_011185366.1\| PREDICTED: odorant receptor 13a-like [Bactrocera cucurbitae] | 1.00E-39 | 37% | 2 | No |
| CL6767.Contig1 | EbalOR43 | 611 | 203 | ref\|XP_011208819.1\| PREDICTED: putative odorant receptor 92a [Bactrocera dorsalis] | 1.00E-14 | 25% | 3 | No |
| Unigene2544 | EbalOR44 | 616 | 191 | ref\|XP_005189143.1\| PREDICTED: odorant receptor 83a [Musca domestica] | 2.00E-23 | 36% | 2 | No |
| Unigene21188 | EbalOR45 | 564 | 163 | gb\|AKI29034.1\| odorant receptor 35a [Bactrocera dorsalis] | 5.00E-12 | 31% | 3 | No |
| Unigene16649 | EbalOR46 | 473 | 157 | ref\|XP_011206229.1\| PREDICTED: odorant receptor 10a [Bactrocera dorsalis] | 6.00E-08 | 30% | 0 | No |
| Unigene14271 | EbalOR47 | 438 | 145 | ref\|XP_001356952.1\|gb\|EAL34018.1\| Or22c [Drosophila pseudoobscura pseudoobscura] | 2.00E-07 | 27% | 2 | No |
| Unigene21382 | EbalOR48 | 422 | 140 | ref\|NP_523454.2\|gb\|AAF51309.2\| odorant receptor 22c [Drosophila melanogaster] | 2.00E-13 | 25% | 2 | No |
| Unigene2242 | EbalOR49 | 627 | 135 | ref\|XP_005182622.1\| PREDICTED: putative odorant receptor 85e [Musca domestica] | 5.00E-38 | 56% | 2 | No |
| Unigene12474 | EbalOR50 | 361 | 120 | ref\|XP_011208819.1\| PREDICTED: putative odorant receptor 92a [Bactrocera dorsalis] | 2.00E-07 | 33% | 2 | No |
| ***E. corollae*** |  |  |  |  |  |  |  |  |
| CL186.Contig1 | EcorOrco | 1950 | 476 | ref\|XP_005175278.1\|odorant receptor coreceptor [Musca domestica] | 1.00E-54 | 87% | 7 | Yes |
| Unigene7263 | EcorOR1 | 1374 | 440 | ref\|XP_011184142.1\| PREDICTED: odorant receptor 83a-like [Bactrocera cucurbitae] | 4.00E-34 | 25% | 6 | No |
| CL6716.Contig1 | EcorOR2 | 1379 | 425 | gb\|AID61215.1\| odorant receptor [Calliphora stygia] | 1.00E-126 | 48% | 7 | Yes |
| Unigene3329 | EcorOR3 | 1392 | 424 | ref\|XP_005180133.1\| PREDICTED: putative odorant receptor 69a, isoform B [Musca domestica] | 1.00E-133 | 50% | 6 | Yes |
| Unigene19189 | EcorOR4 | 1339 | 424 | ref\|XP_011295797.1\| PREDICTED: odorant receptor 13a [Musca domestica] | 1.00E-159 | 51% | 5 | Yes |
| Unigene19077 | EcorOR5 | 1656 | 423 | ref\|XP_011185366.1\| PREDICTED: odorant receptor 13a-like [Bactrocera cucurbitae] | 9.00E-84 | 35% | 7 | Yes |
| Unigene17159 | EcorOR6 | 1315 | 414 | ref\|XP_005178182.1\| PREDICTED: odorant receptor 63a [Musca domestica] | 1.00E-122 | 43% | 8 | Yes |
| CL6486.Contig2 | EcorOR7 | 2067 | 413 | ref\|XP_011185366.1\| PREDICTED: odorant receptor 13a-like [Bactrocera cucurbitae] | 9.00E-63 | 31% | 6 | Yes |
| CL6188.Contig1 | EcorOR8 | 1359 | 405 | ref\|XP_004533437.2\| PREDICTED: odorant receptor 67d-like [Ceratitis capitata] | 1.00E-104 | 42% | 6 | No |
| Unigene15898 | EcorOR9 | 1379 | 405 | ref\|XP_011210110.1\| PREDICTED: odorant receptor 22c [Bactrocera dorsalis] | 5.00E-59 | 29% | 5 | Yes |
| Unigene15973 | EcorOR10 | 1294 | 405 | ref\|XP_005178484.1\| PREDICTED: odorant receptor 10a [Musca domestica] | 6.00E-51 | 30% | 6 | Yes |
| CL3497.Contig1 | EcorOR11 | 1372 | 404 | ref\|XP_005178484.1\| PREDICTED: odorant receptor 10a [Musca domestica] | 1.00E-54 | 30% | 7 | Yes |
| CL4368.Contig1 | EcorOR12 | 1499 | 403 | ref\|XP_005185292.1\| PREDICTED: odorant receptor 74a-like [Musca domestica] | 3.00E-61 | 29% | 5 | Yes |
| CL5507.Contig1 | EcorOR13 | 1393 | 403 | ref\|XP_011200401.1\| PREDICTED: odorant receptor 67c-like [Bactrocera dorsalis] | 1.00E-90 | 39% | 6 | No |
| CL3985.Contig3 | EcorOR14 | 2354 | 402 | ref\|XP_011300122.1\| PREDICTED: odorant receptor 24a-like [Fopius arisanus] | 2.00E-79 | 40% | 5 | Yes |
| CL1193.Contig1 | EcorOR15 | 1421 | 402 | ref\|XP_011210110.1\| PREDICTED: odorant receptor 22c [Bactrocera dorsalis] | 6.00E-47 | 26% | 5 | Yes |
| CL3497.Contig3 | EcorOR16 | 1333 | 401 | ref\|XP_001356952.1\| gb\|EAL34018.1\| Or22c [Drosophila pseudoobscura pseudoobscura] | 3.00E-40 | 26% | 5 | Yes |
| Unigene11158 | EcorOR17 | 1415 | 400 | gb\|AID61213.1\| odorant receptor, partial [Calliphora stygia] | 1.00E-109 | 44% | 6 | Yes |
| CL5741.Contig1 | EcorOR18 | 1762 | 398 | ref\|XP_011179733.1\| PREDICTED: odorant receptor 94a-like [Bactrocera cucurbitae] | 1.00E-108 | 43% | 6 | Yes |
| Unigene15072 | EcorOR19 | 1389 | 398 | ref\|XP_011210110.1\| PREDICTED: odorant receptor 22c [Bactrocera dorsalis] | 1.00E-119 | 45% | 5 | Yes |
| Unigene13937 | EcorOR20 | 1237 | 397 | ref\|XP_005180133.1\| PREDICTED: putative odorant receptor 69a, isoform B [Musca domestica] | 3.00E-27 | 25% | 6 | No |
| CL654.Contig1 | EcorOR21 | 1304 | 393 | ref\|XP_004521076.1\| PREDICTED: odorant receptor 67c-like [Ceratitis capitata] | 1.00E-108 | 43% | 6 | Yes |
| CL6659.Contig1 | EcorOR22 | 1505 | 391 | ref\|XP_011212431.1\| PREDICTED: odorant receptor 49a-like [Bactrocera dorsalis] | 6.00E-61 | 32% | 4 | Yes |
| Unigene7304 | EcorOR23 | 1224 | 391 | gb\|AID61232.1\| odorant receptor [Calliphora stygia] | 1.00E-156 | 59% | 4 | Yes |
| Unigene19150 | EcorOR24 | 1341 | 391 | gb\|AID61221.1\| odorant receptor, partial [Calliphora stygia] | 1.00E-106 | 44% | 6 | Yes |
| Unigene2185 | EcorOR25 | 1479 | 388 | gb\|AID61212.1\| odorant receptor, partial [Calliphora stygia] | 4.00E-93 | 39% | 6 | Yes |
| CL5624.Contig2 | EcorOR26 | 1316 | 386 | ref\|XP_011208898.1\| PREDICTED: odorant receptor 7a-like [Bactrocera dorsalis] | 6.00E-74 | 35% | 5 | No |
| CL5290.Contig1 | EcorOR27 | 1670 | 384 | gb\|AID61224.1\| odorant receptor [Calliphora stygia] | 1.00E-125 | 49% | 6 | Yes |
| Unigene19174 | EcorOR28 | 1235 | 384 | ref\|XP_011211752.1\| PREDICTED: odorant receptor 94a [Bactrocera dorsalis] | 7.00E-79 | 36% | 5 | Yes |
| Unigene9131 | EcorOR29 | 1356 | 383 | ref\|XP_011203703.1\| PREDICTED: odorant receptor 67d-like [Bactrocera dorsalis] | 1.00E-110 | 43% | 6 | Yes |
| CL5908.Contig1 | EcorOR30 | 1336 | 381 | ref\|XP_011208819.1\| PREDICTED: putative odorant receptor 92a [Bactrocera dorsalis] | 2.00E-63 | 31% | 6 | Yes |
| CL5278.Contig2 | EcorOR31 | 1206 | 380 | gb\|AID61202.1\| odorant receptor [Calliphora stygia] | 1.00E-137 | 52% | 5 | No |
| CL3926.Contig1 | EcorOR32 | 1234 | 379 | ref\|XP_011684724.1\| PREDICTED: odorant receptor 82a-like [Wasmannia auropunctata] | 2.00E-12 | 22% | 6 | No |
| Unigene14295 | EcorOR33 | 1227 | 378 | gb\|EFA05710.1\| odorant receptor 73 [Tribolium castaneum] | 4.00E-11 | 22% | 6 | No |
| Unigene15962 | EcorOR34 | 1163 | 378 | ref\|XP_011210110.1\| PREDICTED: odorant receptor 22c [Bactrocera dorsalis] | 1.00E-41 | 25% | 5 | No |
| Unigene1764 | EcorOR35 | 1204 | 372 | gb\|AID61210.1\| odorant receptor [Calliphora stygia] | 1.00E-151 | 56% | 7 | Yes |
| CL4681.Contig2 | EcorOR36 | 1485 | 371 | gb\|AID61215.1\| odorant receptor [Calliphora stygia] | 1.00E-176 | 65% | 4 | Yes |
| Unigene5973 | EcorOR37 | 1421 | 349 | gb\|AID61211.1\| odorant receptor [Calliphora stygia] | 4.00E-89 | 44% | 6 | No |
| CL3497.Contig2 | EcorOR38 | 1262 | 332 | ref\|XP_011210110.1\| PREDICTED: odorant receptor 22c [Bactrocera dorsalis] | 4.00E-45 | 27% | 4 | No |
| Unigene8086 | EcorOR39 | 869 | 289 | ref\|XP_012157159.1\| PREDICTED: odorant receptor 2a-like [Ceratitis capitata] | 1.00E-66 | 37% | 3 | No |
| Unigene7951 | EcorOR40 | 2677 | 287 | ref\|XP_011185366.1\| PREDICTED: odorant receptor 13a-like [Bactrocera cucurbitae] | 4.00E-48 | 37% | 2 | Yes |
| Unigene24500 | EcorOR41 | 206 | 109 | ref\|XP_005190411.2\| PREDICTED: odorant receptor 85c-like [Musca domestica] | 2.00E-38 | 62% | 1 | No |

Table S2-2. Unigenes of candidate gustatory receptors in *E. balteatus* and *E. corollae*

| Unigene reference | Gene name | Length (nt) | ORF (aa) | Blastx best hit (Reference/Name/Species) | Evalue | Identity | TMD (No.) | Full length |
| --- | --- | --- | --- | --- | --- | --- | --- | --- |
| ***E. balteatus*** |  |  |  |  |  |  |  |  |
| Unigene7175 | EbalGR1 | 1514 | 455 | gb\|AID61256.1\| gustatory receptor [Calliphora stygia] | 1.00E-178 | 63% | 7 | Yes |
| Unigene15398 | EbalGR2 | 1526 | 444 | gb\|AFH96948.1\| gustatory receptor 1 [Musca domestica] | 0 | 81% | 6 | Yes |
| Unigene10693 | EbalGR3 | 1396 | 444 | gb\|AID61262.1\| gustatory receptor [Calliphora stygia] | 0 | 68% | 7 | Yes |
| Unigene407 | EbalGR4 | 568 | 173 | gb\|AID61254.1\| gustatory receptor, partial [Calliphora stygia] | 9.00E-94 | 95% | 3 | No |
| Unigene10807 | EbalGR5 | 514 | 140 | ref\|XP_011181424.1\| PREDICTED: gustatory receptor for sugar taste 64a [Bactrocera cucurbitae] | 9.00E-19 | 47% | 2 | No |
| Unigene5239 | EbalGR6 | 374 | 124 | ref\|XP_001353665.1\| Gr64a [Drosophila pseudoobscura pseudoobscura] | 6.00E-61 | 74% | 1 | No |
| Unigene16862 | EbalGR7 | 367 | 121 | ref\|XP_011180395.1\| PREDICTED: putative gustatory receptor 28a [Bactrocera cucurbitae] | 6.00E-08 | 29% | 3 | No |
| Unigene25792 | EbalGR8 | 347 | 115 | ref\|XP_012217698.1\| PREDICTED: gustatory receptor for sugar taste 64f-like [Linepithema humile] | 2.00E-15 | 34% | 1 | No |
| Unigene31910 | EbalGR9 | 266 | 88 | gb\|AID61265.1\| gustatory receptor [Calliphora stygia] | 6.00E-28 | 62% | 0 | No |
| Unigene1040 | EbalGR10 | 257 | 85 | ref\|XP_001353665.1\|gb\|EAL31179.1\| Gr64a [Drosophila pseudoobscura pseudoobscura] | 2.00E-16 | 45% | 0 | No |
| Unigene2063 | EbalGR11 | 379 | 75 | gb\|AID61257.1\| gustatory receptor, partial [Calliphora stygia] | 6.00E-37 | 84% | 0 | No |
| Unigene25996 | EbalGR12 | 457 | 75 | ref\|XP_012530688.1\| PREDICTED: gustatory receptor for sugar taste 64f-like isoform X3 [Monomorium pharaonis] | 8.00E-15 | 48% | 1 | No |
| Unigene34382 | EbalGR13 | 221 | 73 | ref\|NP_995643.1\| gb\|AAS64648.1\|gustatory receptor 28b, isoform B [Drosophila melanogaster] | 2.00E-26 | 67% | 0 | No |
| Unigene26675 | EbalGR14 | 202 | 66 | ref\|XP_005175753.1\| PREDICTED: gustatory receptor for sugar taste 64e, partial [Musca domestica] | 3.00E-28 | 75% | 0 | No |
| ***E. corollae*** |  |  |  |  |  |  |  |  |
| Unigene9155 | EcorGR1 | 1713 | 455 | gb\|AID61256.1\| gustatory receptor [Calliphora stygia] | 0 | 64% | 7 | Yes |
| CL5822.Contig1 | EcorGR2 | 1837 | 444 | gb\|AFH96948.1\| gustatory receptor 1 [Musca domestica] | 0 | 82% | 6 | Yes |
| CL6744.Contig1 | EcorGR3 | 1817 | 444 | gb\|AID61262.1\| gustatory receptor [Calliphora stygia] | 0 | 68% | 7 | Yes |
| Unigene2248 | EcorGR4 | 1332 | 442 | ref\|XP_011180327.1\| PREDICTED: putative gustatory receptor 28b [Bactrocera cucurbitae] | 0 | 62% | 8 | No |
| Unigene13906 | EcorGR5 | 1147 | 381 | ref\|NP_724332.1\| gb\|AAN11117.1\|gustatory receptor 39a, isoform A [Drosophila melanogaster] | 6.00E-45 | 31% | 7 | No |
| Unigene5145 | EcorGR6 | 1448 | 372 | ref\|XP_012160377.1\| PREDICTED: putative gustatory receptor 39b [Ceratitis capitata] | 4.00E-34 | 25% | 6 | Yes |
| Unigene15056 | EcorGR7 | 1630 | 372 | ref\|XP_012160377.1\| PREDICTED: putative gustatory receptor 39b [Ceratitis capitata] | 2.00E-40 | 28% | 6 | Yes |
| Unigene13918 | EcorGR8 | 961 | 266 | ref\|XP_011181427.1\| PREDICTED: gustatory receptor for sugar taste 64c-like [Bactrocera cucurbitae] | 2.00E-82 | 45% | 3 | Yes |
| Unigene23213 | EcorGR9 | 392 | 130 | emb\|CBA14491.1\| gustatory receptor 98a [Drosophila simulans] | 1.00E-05 | 24% | 0 | No |
| Unigene30040 | EcorGR10 | 388 | 129 | gb\|AID61265.1\| gustatory receptor [Calliphora stygia] | 2.00E-50 | 63% | 2 | No |
| Unigene31845 | EcorGR11 | 317 | 105 | ref\|XP_005186933.1\| PREDICTED: gustatory receptor 5a for trehalose, partial [Musca domestica] | 1.00E-34 | 55% | 1 | No |
| Unigene30721 | EcorGR12 | 312 | 103 | gb\|AID61265.1\| gustatory receptor [Calliphora stygia] | 7.00E-42 | 64% | 2 | No |
| Unigene13035 | EcorGR13 | 289 | 79 | ref\|XP_004533711.1\| PREDICTED: putative gustatory receptor 98b [Ceratitis capitata] | 2.00E-18 | 54% | 1 | No |
| Unigene787 | EcorGR14 | 213 | 70 | gb\|AID61271.1\| gustatory receptor, partial [Calliphora stygia] | 2.00E-32 | 75% | 0 | No |
| Unigene27047 | EcorGR15 | 205 | 67 | ref\|XP_011181427.1\| PREDICTED: gustatory receptor for sugar taste 64c-like [Bactrocera cucurbitae] | 2.00E-10 | 45% | 1 | No |
| Unigene24798 | EcorGR16 | 200 | 66 | ref\|XP_011207478.1\| PREDICTED: gustatory receptor for bitter taste 66a [Bactrocera dorsalis] | 7.00E-36 | 93% | 0 | No |

Table S2-3. Unigenes of candidate ionotropic receptors in *E. balteatus* and *E. corollae*

| Unigene reference | Gene name | Length (nt) | ORF (aa) | Blastx best hit (Reference/Name/Species) | Evalue | Identity | TMD  (No.) | Full length |
| --- | --- | --- | --- | --- | --- | --- | --- | --- |
| ***E. balteatus*** |  |  |  |  |  |  |  |  |
| Unigene2332 | EbalIR21a | 2666 | 887 | ref\|NP_001097043.1\| gb\|AAF51569.2\|ionotropic receptor 21a [Drosophila melanogaster] | 0 | 62% | 5 | No |
| Unigene17164 | EbalIR25a | 3136 | 939 | ref\|XP_011178452.1\| PREDICTED: glutamate receptor 3 [Bactrocera cucurbitae] | 0 | 81% | 3 | Yes |
| Unigene18037 | EbalIR31a.1 | 676 | 196 | gb\|AID61274.1\| ionotropic receptor, partial [Calliphora stygia] | 6.00E-62 | 53% | 1 | No |
| Unigene13658 | EbalIR31a.2 | 2512 | 637 | ref\|NP_001260346.1\| gb\|AGB92881.1\| ionotropic receptor 31a, isoform C [Drosophila melanogaster] | 0 | 46% | 4 | No |
| Unigene10931 | EbalIR31a.3 | 1009 | 311 | ref\|NP_723585.2\| gb\|AAN10751.2\| ionotropic receptor 31a, isoform D [Drosophila melanogaster] | 1.00E-81 | 44% | 0 | No |
| Unigene5153 | EbalIR31a.4 | 1933 | 628 | ref\|NP_001260346.1\| gb\|AGB92881.1\| ionotropic receptor 31a, isoform C [Drosophila melanogaster] | 0 | 49% | 3 | No |
| Unigene11710 | EbalIR40a | 2120 | 706 | gb\|AID61275.1\| ionotropic receptor [Calliphora stygia] | 0 | 58% | 3 | No |
| Unigene14034 | EbalIR64a | 3099 | 937 | gb\|AID61277.1\| ionotropic receptor, partial [Calliphora stygia] | 1.00E-121 | 62% | 1 | No |
| Unigene7580 | EbalIR68a | 337 | 111 | ref\|NP_001287031.1\| gb\|AHN58056.1\| ionotropic receptor 68a, isoform B [Drosophila melanogaster] | 2.00E-28 | 50% | 1 | No |
| Unigene528 | EbalIR75d | 1974 | 647 | gb\|AKI28987.1\| ionotropic receptor 75d [Bactrocera dorsalis] | 0 | 54% | 3 | No |
| CL2730.Contig1 | EbalIR75l | 1947 | 508 | dbj\|BAR64808.1\| ionotropic receptor [Ostrinia furnacalis] | 1.00E-137 | 44% | 3 | No |
| CL3965.Contig1 | EbalIR76b | 2286 | 617 | ref\|NP_649176.1\| gb\|AAF49071.1\| ionotropic receptor 76b [Drosophila melanogaster] | 0 | 60% | 3 | Yes |
| Unigene12526 | EbalIR84a | 2399 | 668 | ref\|XP_011193628.1\| PREDICTED: glutamate receptor 1 [Bactrocera cucurbitae] | 1.00E-128 | 37% | 4 | No |
| Unigene12384 | EbalIR8a | 2766 | 885 | gb\|AID61272.1\| ionotropic receptor [Calliphora stygia] | 0 | 57% | 3 | Yes |
| Unigene1939 | EbalIR92a.1 | 2017 | 652 | gb\|AKI28990.1\| ionotropic receptor 92a [Bactrocera dorsalis] | 1.00E-177 | 40% | 3 | Yes |
| Unigene12098 | EbalIR92a.2 | 2003 | 641 | gb\|AKI28990.1\| ionotropic receptor 92a [Bactrocera dorsalis] | 1.00E-174 | 41% | 3 | Yes |
| Unigene6392 | EbalIR92a.3 | 934 | 299 | gb\|AID61282.1\| ionotropic receptor, partial [Calliphora stygia] | 3.00E-82 | 47% | 3 | No |
| Unigene12467 | EbalIR93a | 536 | 178 | gb\|AID61283.1\| ionotropic receptor, partial [Calliphora stygia] | 1.00E-72 | 70% | 1 | No |
| CL397.Contig2 | EbalIR1 | 1992 | 640 | gb\|AID61276.1\| ionotropic receptor [Calliphora stygia] | 1.00E-164 | 40% | 3 | Yes |
| Unigene56 | EbalIR2 | 1849 | 616 | ref\|NP_649013.3\| gb\|AAF49299.3\| ionotropic receptor 75c [Drosophila melanogaster] | 1.00E-152 | 38% | 4 | No |
| CL4913.Contig3 | EbalIR3 | 1735 | 560 | ref\|NP_729609.1\| gb\|AAN11914.1\| ionotropic receptor 67c [Drosophila melanogaster] | 4.00E-39 | 23% | 3 | No |
| CL4118.Contig1 | EbalIR4 | 1848 | 506 | ref\|NP_001097885.2\|gb\|AAF56134.4\| ionotropic receptor 94e [Drosophila melanogaster] | 4.00E-51 | 28% | 2 | Yes |
| CL405.Contig1 | EbalIR5 | 1278 | 425 | ref\|NP_725440.1\| gb\|AAM68524.1\| ionotropic receptor 51b [Drosophila melanogaster] | 7.00E-17 | 22% | 3 | No |
| Unigene487 | EbalIR6 | 971 | 323 | ref\|NP_001097885.2\| gb\|AAF56134.4\| ionotropic receptor 94e [Drosophila melanogaster] | 6.00E-46 | 31% | 3 | No |
| Unigene18834 | EbalIR7 | 905 | 301 | ref\|NP_001097885.2\| gb\|AAF56134.4\| ionotropic receptor 94e [Drosophila melanogaster] | 5.00E-38 | 32% | 2 | No |
| CL2647.Contig1 | EbalIR8 | 807 | 268 | ref\|NP_001097885.2\|gb\|AAF56134.4\| ionotropic receptor 94e [Drosophila melanogaster] | 3.00E-26 | 29 | 2 | No |
| Unigene27359 | EbalIR9 | 646 | 215 | ref\|NP_001097885.2\|gb\|AAF56134.4\| ionotropic receptor 94e [Drosophila melanogaster] | 7.00E-26 | 34% | 1 | No |
| Unigene22629 | EbalIR10 | 662 | 213 | gb\|AKI28986.1\| ionotropic receptor 41a [Bactrocera dorsalis] | 5.00E-58 | 44% | 1 | No |
| Unigene15067 | EbalIR11 | 598 | 199 | ref\|NP_725440.1\|gb\|AAM68524.1\| ionotropic receptor 51b [Drosophila melanogaster] | 2.00E-12 | 29% | 0 | No |
| CL5180.Contig1 | EbalIR12 | 567 | 188 | ref\|NP_001097885.2\|gb\|AAF56134.4\| ionotropic receptor 94e [Drosophila melanogaster] | 3.00E-26 | 32% | 0 | No |
| Unigene23687 | EbalIR13 | 560 | 180 | ref\|NP_608456.1\| gb\|AAF50808.1\|ionotropic receptor 20a [Drosophila melanogaster] | 1.00E-19 | 28% | 1 | No |
| Unigene9613 | EbalIR14 | 529 | 175 | ref\|NP_001138099.1\| gb\|ACL83555.1\| ionotropic receptor 94d [Drosophila melanogaster] | 1.00E-21 | 29% | 2 | No |
| ***E. corollae*** |  |  |  |  |  |  |  |  |
| Unigene9380 | EcorIR21a | 2771 | 897 | ref\|NP_001097043.1\| gb\|AAF51569.2\| ionotropic receptor 21a [Drosophila melanogaster] | 0 | 60% | 3 | Yes |
| CL2001.Contig1 | EcorIR25a | 3098 | 939 | ref\|XP_011178452.1\| PREDICTED: glutamate receptor 3 [Bactrocera cucurbitae] | 0 | 81% | 3 | Yes |
| CL3624.Contig1 | EcorIR31a.1 | 2513 | 635 | ref\|NP_001260346.1\| gb\|AGB92881.1\| ionotropic receptor 31a, isoform C [Drosophila melanogaster] | 1.00E-178 | 42% | 3 | Yes |
| Unigene18014 | EcorIR31a.2 | 1890 | 609 | ref\|NP_001260346.1\| gb\|AGB92881.1\|ionotropic receptor 31a, isoform C [Drosophila melanogaster] | 0 | 50% | 3 | No |
| Unigene3974 | EcorIR40a | 2154 | 717 | gb\|AID61275.1\| ionotropic receptor [Calliphora stygia] | 0 | 56% | 3 | No |
| Unigene16097 | EcorIR64a | 3928 | 1146 | gb\|AID61277.1\| ionotropic receptor, partial [Calliphora stygia] | 1.00E-117 | 60% | 3 | Yes |
| Unigene5347 | EcorIR75d | 2198 | 674 | gb\|AKI28987.1\| ionotropic receptor 75d [Bactrocera dorsalis] | 0 | 62% | 4 | Yes |
| CL2212.Contig2 | EcorIR75l | 2110 | 645 | ref\|XP_975640.3\| PREDICTED: probable glutamate receptor [Tribolium castaneum] | 1.00E-141 | 44% | 4 | Yes |
| CL2333.Contig2 | EcorIR76b | 2290 | 617 | ref\|XP_011290996.1\| PREDICTED: glutamate receptor 2 [Musca domestica] | 0 | 59% | 3 | Yes |
| CL2773.Contig2 | EcorIR84a | 2253 | 645 | ref\|XP_011193628.1\| PREDICTED: glutamate receptor 1 [Bactrocera cucurbitae] | 1.00E-133 | 36% | 4 | Yes |
| CL7020.Contig1 | EcorIR8a | 2820 | 885 | gb\|AID61272.1\| ionotropic receptor [Calliphora stygia] | 0 | 56% | 4 | Yes |
| CL2034.Contig1 | EcorIR92a.1 | 2043 | 645 | gb\|AKI28990.1\| ionotropic receptor 92a [Bactrocera dorsalis] | 0 | 42% | 2 | Yes |
| CL6399.Contig1 | EcorIR92a.2 | 2015 | 640 | gb\|AKI28990.1\| ionotropic receptor 92a [Bactrocera dorsalis] | 1.00E-180 | 42% | 3 | Yes |
| Unigene7934 | EcorIR93a.1 | 1339 | 446 | ref\|NP_650924.3\| gb\|AAF55817.3\| ionotropic receptor 93a [Drosophila melanogaster] | 0 | 56% | 3 | No |
| Unigene5803 | EcorIR93a.2 | 474 | 135 | ref\|XP_011293092.1\| PREDICTED: glutamate receptor ionotropic, delta-1 [Musca domestica] | 4.00E-51 | 70% | 1 | No |
| Unigene7956 | EcorIR93a.3 | 885 | 291 | ref\|XP_011293092.1\| PREDICTED: glutamate receptor ionotropic, delta-1 [Musca domestica] | 1.00E-102 | 53% | 0 | No |
| Unigene5370 | EcorIR1 | 2234 | 642 | gb\|AID61276.1\| ionotropic receptor [Calliphora stygia] | 1.00E-163 | 40% | 3 | Yes |
| Unigene9172 | EcorIR2 | 1947 | 621 | ref\|XP_011296250.1\| PREDICTED: glutamate receptor-like [Musca domestica] | 1.00E-162 | 41% | 4 | Yes |
| CL2693.Contig1 | EcorIR3 | 2081 | 592 | ref\|NP_729609.1\| gb\|AAN11914.1\| ionotropic receptor 67c [Drosophila melanogaster] | 9.00E-40 | 25% | 5 | Yes |
| Unigene317 | EcorIR4 | 1733 | 577 | ref\|NP_611432.1\| gb\|AAF57537.1\| ionotropic receptor 56d [Drosophila melanogaster] | 2.00E-29 | 24% | 3 | No |
| Unigene19061 | EcorIR5 | 1764 | 574 | ref\|NP_611432.1\| gb\|AAF57537.1\| ionotropic receptor 56d [Drosophila melanogaster] | 3.00E-28 | 23% | 3 | No |
| Unigene2056 | EcorIR6 | 1428 | 466 | ref\|NP_001097885.2\| gb\|AAF56134.4\|ionotropic receptor 94e [Drosophila melanogaster] | 1.00E-101 | 38% | 3 | No |
| CL109.Contig3 | EcorIR7 | 1930 | 343 | gb\|AKI28986.1\| ionotropic receptor 41a [Bactrocera dorsalis] | 7.00E-84 | 40% | 3 | No |

Table S2-4. Unigenes of candidate odorant binding proteins in *E. balteatus* and *E. corollae*

| Unigene reference | Gene name | Length (nt) | ORF (aa) | Blastx best hit (Reference/Name/Species) | Evalue | Identity | Signal peptide | Full length |
| --- | --- | --- | --- | --- | --- | --- | --- | --- |
| ***E. balteatus*** |  |  |  |  |  |  |  |  |
| Unigene3972 | EbalOBP1 | 855 | 260 | gb\|AKI29006.1\| odorant binding protein 50c [Bactrocera dorsalis] | 1.00E-44 | 37% | 19 | Yes |
| Unigene7173 | EbalOBP2 | 1103 | 253 | gb\|AIE43941.1\| gb\|AIE43949.1\|odorant-binding protein 29, partial [Lutzomyia longipalpis] | 7.00E-52 | 39% | 24 | Yes |
| Unigene10986 | EbalOBP3 | 692 | 208 | ref\|XP_002138644.1\| gb\|EDY69202.1\|Odorant-binding protein 49a [Drosophila pseudoobscura pseudoobscura] | 5.00E-17 | 27% | 23 | Yes |
| Unigene13257 | EbalOBP4 | 1702 | 194 | gb\|AID61316.1\| odorant binding protein [Calliphora stygia] | 4.00E-56 | 53% | 27 | Yes |
| Unigene6082 | EbalOBP5 | 603 | 162 | >ref\|XP_004535942.1\| PREDICTED: general odorant-binding protein 99a [Ceratitis capitata] | 4.00E-10 | 28% | 26 | No |
| Unigene22348 | EbalOBP6 | 562 | 157 | >dbj\|BAN59722.1\| odorant-binding protein [Delia antiqua] | 2.00E-24 | 34% | 19 | Yes |
| CL142.Contig1 | EbalOBP7 | 568 | 156 | ref\|XP_001360913.1\| gb\|EAL25488.1\|Odorant-binding protein 44a [Drosophila pseudoobscura pseudoobscura] | 1.00E-14 | 32% | 15 | Yes |
| CL142.Contig3 | EbalOBP8 | 536 | 156 | ref\|XP_001360913.1\| gb\|EAL25488.1\|Odorant-binding protein 44a [Drosophila pseudoobscura pseudoobscura] | 3.00E-15 | 31% | 15 | Yes |
| CL142.Contig2 | EbalOBP9 | 495 | 155 | ref\|XP_011210425.1\| PREDICTED: general odorant-binding protein 99a-like [Bactrocera dorsalis] | 4.00E-15 | 33% | 16 | Yes |
| Unigene17855 | EbalOBP10 | 875 | 153 | gb\|AID61309.1\| odorant binding protein [Calliphora stygia] | 1.00E-12 | 36% | 19 | Yes |
| Unigene2526 | EbalOBP11 | 608 | 151 | gb\|AKI29024.1\| odorant binding protein 99c-2 [Bactrocera dorsalis] | 8.00E-09 | 29% | 15 | Yes |
| Unigene17669 | EbalOBP12 | 588 | 150 | ref\|XP_011210417.1\| PREDICTED: general odorant-binding protein 99a-like [Bactrocera dorsalis] | 3.00E-43 | 55% | 16 | Yes |
| Unigene8698 | EbalOBP13 | 571 | 149 | ref\|XP_011184805.1\| PREDICTED: general odorant-binding protein 84a-like [Bactrocera cucurbitae] | 7.00E-45 | 56% | 24 | Yes |
| Unigene15335 | EbalOBP14 | 554 | 149 | gb\|AKI28997.1\| odorant binding protein 8a [Bactrocera dorsalis] | 2.00E-41 | 49% | 17 | Yes |
| Unigene19987 | EbalOBP15 | 589 | 149 | gb\|AID61318.1\| odorant binding protein [Calliphora stygia] | 3.00E-11 | 33% | 15 | Yes |
| CL456.Contig1 | EbalOBP16 | 632 | 148 | gb\|AID61318.1\| odorant binding protein [Calliphora stygia] | 1.00E-10 | 30% | 15 | Yes |
| CL118.Contig3 | EbalOBP17 | 1437 | 147 | gb\|AKI28996.1\| odorant binding protein lush [Bactrocera dorsalis] | 4.00E-58 | 73% | 24 | Yes |
| Unigene14055 | EbalOBP18 | 532 | 146 | ref\|XP_005179081.1\| PREDICTED: general odorant-binding protein 99a [Musca domestica] | 4.00E-12 | 29% | 15 | Yes |
| Unigene15287 | EbalOBP19 | 681 | 146 | gb\|ACY93401.1\| gb\|ACY93453.1\|odorant binding protein 19b [Drosophila melanogaster] | 2.00E-23 | 38% | 19 | Yes |
| Unigene15591 | EbalOBP20 | 874 | 146 | ref\|NP_001295333.1\| emb\|CDJ79887.1\|general odorant-binding protein 83a precursor [Ceratitis capitata] | 1.00E-64 | 74% | 20 | Yes |
| CL1856.Contig1 | EbalOBP21 | 1324 | 146 | ref\|XP_011210420.1\| PREDICTED: general odorant-binding protein 99a [Bactrocera dorsalis] | 2.00E-49 | 58% | 16 | Yes |
| CL1116.Contig1 | EbalOBP22 | 968 | 145 | ref\|XP_011187213.1\| PREDICTED: general odorant-binding protein 19a [Bactrocera cucurbitae] | 5.00E-62 | 66% | 23 | Yes |
| Unigene7463 | EbalOBP23 | 1542 | 144 | gb\|AID61303.1\| odorant binding protein [Calliphora stygia] | 4.00E-70 | 68% | 20 | Yes |
| Unigene4118 | EbalOBP24 | 745 | 143 | ref\|XP_011198820.1\| PREDICTED: general odorant-binding protein 28a-like [Bactrocera dorsalis] | 7.00E-34 | 43% | 18 | Yes |
| Unigene11016 | EbalOBP25 | 627 | 142 | ref\|XP_005179081.1\| PREDICTED: general odorant-binding protein 99a [Musca domestica] | 7.00E-63 | 67% | 18 | Yes |
| Unigene19952 | EbalOBP26 | 592 | 142 | ref\|XP_011205204.1\| PREDICTED: general odorant-binding protein 99a [Bactrocera dorsalis] | 6.00E-16 | 33% | 17 | Yes |
| CL647.Contig1 | EbalOBP27 | 1872 | 141 | ref\|NP_611442.1\| gb\|AAF57522.1\|Odorant-binding protein 56a [Drosophila melanogaster] | 5.00E-14 | 39% | NO | No |
| CL4957.Contig1 | EbalOBP28 | 489 | 138 | ref\|XP_001361436.1\| gb\|EAL26014.1\| Odorant-binding protein 56a [Drosophila pseudoobscura pseudoobscura] | 2.00E-17 | 36% | 19 | Yes |
| Unigene8712 | EbalOBP29 | 604 | 138 | dbj\|BAM78536.1\| odorant-binding protein [Phormia regina] | 4.00E-12 | 32% | 23 | Yes |
| Unigene6183 | EbalOBP30 | 465 | 137 | ref\|XP_001361436.1\| gb\|EAL26014.1\| Odorant-binding protein 56a [Drosophila pseudoobscura pseudoobscura] | 6.00E-14 | 47% | 20 | Yes |
| CL1460.Contig1 | EbalOBP31 | 1313 | 137 | gb\|AID61302.1\| odorant binding protein [Calliphora stygia] | 7.00E-32 | 43% | NO | Yes |
| Unigene13083 | EbalOBP32 | 429 | 135 | gb\|AID61309.1\| odorant binding protein [Calliphora stygia] | 6.00E-11 | 31% | 18 | Yes |
| Unigene1495 | EbalOBP33 | 516 | 132 | ref\|XP_001361436.1\| gb\|EAL26014.1\| Odorant-binding protein 56a [Drosophila pseudoobscura pseudoobscura] | 6.00E-20 | 40% | 18 | Yes |
| CL435.Contig1 | EbalOBP34 | 471 | 131 | dbj\|BAN59721.1\| odorant-binding protein [Delia antiqua] | 3.00E-19 | 39% | 25 | Yes |
| Unigene17635 | EbalOBP35 | 500 | 130 | ref\|XP_001361436.1\| gb\|EAL26014.1\| Odorant-binding protein 56a [Drosophila pseudoobscura pseudoobscura] | 1.00E-19 | 38% | 18 | Yes |
| Unigene4399 | EbalOBP36 | 389 | 129 | ref\|NP_611442.1\| gb\|AAF57522.1\| Odorant-binding protein 56a [Drosophila melanogaster] | 1.00E-21 | 40% | 20 | No |
| Unigene12954 | EbalOBP37 | 508 | 129 | ref\|XP_011197304.1\| PREDICTED: general odorant-binding protein 56h-like [Bactrocera dorsalis] | 5.00E-12 | 32% | 18 | Yes |
| Unigene18341 | EbalOBP38 | 496 | 129 | emb\|CCD17841.1\| putative odorant binding protein 72 [Nasonia vitripennis] | 7.00E-08 | 27% | 19 | Yes |
| Unigene20600 | EbalOBP39 | 907 | 129 | dbj\|BAM78536.1\| odorant-binding protein [Phormia regina] | 2.00E-13 | 34% | 18 | Yes |
| Unigene1532 | EbalOBP40 | 424 | 126 | ref\|XP_011180831.1\| PREDICTED: general odorant-binding protein 56h-like [Bactrocera cucurbitae] | 2.00E-10 | 31% | 18 | Yes |
| Unigene5444 | EbalOBP41 | 402 | 126 | gb\|AID61309.1\| odorant binding protein [Calliphora stygia] | 3.00E-13 | 31% | 20 | No |
| CL738.Contig3 | EbalOBP42 | 739 | 124 | ref\|NP_611442.1\| gb\|AAF57522.1\|Odorant-binding protein 56a [Drosophila melanogaster] | 4.00E-15 | 39% | 18 | Yes |
| Unigene4438 | EbalOBP43 | 359 | 119 | dbj\|BAM78536.1\| odorant-binding protein [Phormia regina] | 3.00E-06 | 28% | 27 | No |
| Unigene20647 | EbalOBP44 | 405 | 112 | gb\|ADG96061.1\| putative odorant binding protein [Stomoxys calcitrans] | 6.00E-14 | 32% | NO | No |
| CL3008.Contig2 | EbalOBP45 | 303 | 91 | ref\|XP_011205204.1\| PREDICTED: general odorant-binding protein 99a [Bactrocera dorsalis] | 2.00E-07 | 35% | NO | No |
| Unigene26622 | EbalOBP46 | 239 | 79 | gb\|AID61306.1\| odorant binding protein, partial [Calliphora stygia] | 4.00E-07 | 32% | NO | No |
| Unigene26361 | EbalOBP47 | 220 | 73 | gb\|AID61309.1\| odorant binding protein [Calliphora stygia] | 1.00E-12 | 47% | NO | No |
| CL1116.Contig2 | EbalOBP48 | 217 | 59 | gb\|AKI28998.1\| odorant binding protein 19a [Bactrocera dorsalis] | 3.00E-12 | 75% | 23 | No |
| Unigene23654 | EbalOBP49 | 216 | 45 | gb\|AGM37951.1\| pheromone binding protein, partial [Hylamorpha elegans] | 2.00E-07 | 42% | NO | No |
| ***E. corollae*** |  |  |  |  |  |  |  |  |
| CL3115.Contig1 | EcorOBP1 | 976 | 270 | gb\|AIE43918.1\| gb\|AIE43919.1\|odorant-binding protein 29, partial [Lutzomyia longipalpis] | 3.00E-46 | 35% | 26 | Yes |
| CL5936.Contig1 | EcorOBP2 | 922 | 252 | gb\|AKI29006.1\| odorant binding protein 50c [Bactrocera dorsalis] | 3.00E-41 | 34% | 18 | Yes |
| Unigene11838 | EcorOBP3 | 711 | 218 | gb\|AKI29006.1\| odorant binding protein 50c [Bactrocera dorsalis] | 5.00E-42 | 36% | NO | No |
| CL4775.Contig1 | EcorOBP4 | 720 | 203 | ref\|XP_002138644.1\| gb\|EDY69202.1\|Odorant-binding protein 49a [Drosophila pseudoobscura pseudoobscura] | 3.00E-27 | 33% | 23 | Yes |
| Unigene27956 | EcorOBP5 | 506 | 158 | gb\|AID61312.1\| odorant binding protein, partial [Calliphora stygia] | 4.00E-45 | 62% | NO | No |
| Unigene15796 | EcorOBP6 | 637 | 156 | ref\|XP_004535942.1\| PREDICTED: general odorant-binding protein 99a [Ceratitis capitata] | 2.00E-16 | 36% | 17 | Yes |
| CL2290.Contig1 | EcorOBP7 | 1075 | 155 | emb\|CBA11305.1\| odorant binding protein 1 [Glossina morsitans morsitans] | 6.00E-12 | 35% | 16 | Yes |
| Unigene8201 | EcorOBP8 | 565 | 151 | gb\|AKI29022.1\| odorant binding protein 99a [Bactrocera dorsalis] | 5.00E-14 | 32% | 19 | Yes |
| CL1588.Contig1 | EcorOBP9 | 727 | 149 | ref\|XP_011184805.1\| PREDICTED: general odorant-binding protein 84a-like [Bactrocera cucurbitae] | 2.00E-42 | 53% | 18 | Yes |
| Unigene19765 | EcorOBP10 | 679 | 148 | gb\|AKI29022.1\| odorant binding protein 99a [Bactrocera dorsalis] | 2.00E-34 | 50% | 16 | Yes |
| Unigene11759 | EcorOBP11 | 522 | 147 | gb\|AKI28997.1\| odorant binding protein 8a [Bactrocera dorsalis] | 6.00E-46 | 47% | 18 | Yes |
| CL6216.Contig1 | EcorOBP12 | 1246 | 146 | ref\|NP_001295333.1\|emb\|CDJ79887.1\| general odorant-binding protein 83a precursor [Ceratitis capitata] | 4.00E-64 | 65% | 20 | Yes |
| Unigene19727 | EcorOBP13 | 572 | 146 | ref\|XP_004521186.1\| PREDICTED: general odorant-binding protein 99a [Ceratitis capitata] | 1.00E-54 | 65% | 16 | Yes |
| Unigene28185 | EcorOBP14 | 560 | 146 | ref\|XP_011176687.1\| PREDICTED: general odorant-binding protein lush isoform X1 [Bactrocera cucurbitae] | 3.00E-63 | 71% | 23 | Yes |
| CL4124.Contig1 | EcorOBP15 | 925 | 145 | gb\|AID61303.1\| odorant binding protein [Calliphora stygia] | 7.00E-72 | 76% | 21 | Yes |
| CL657.Contig2 | EcorOBP16 | 2134 | 145 | ref\|XP_011187213.1\| PREDICTED: general odorant-binding protein 19a [Bactrocera cucurbitae] | 2.00E-57 | 64% | 23 | Yes |
| CL1650.Contig1 | EcorOBP17 | 654 | 143 | ref\|XP_004525016.1\| PREDICTED: general odorant-binding protein 28a [Ceratitis capitata] | 4.00E-28 | 40% | 24 | Yes |
| Unigene1877 | EcorOBP18 | 507 | 142 | ref\|XP_005179081.1\| PREDICTED: general odorant-binding protein 99a [Musca domestica] | 3.00E-63 | 66% | 18 | Yes |
| Unigene13372 | EcorOBP19 | 560 | 139 | ref\|XP_004517804.1\| PREDICTED: general odorant-binding protein 56h [Ceratitis capitata] | 2.00E-08 | 27% | 21 | Yes |
| Unigene13596 | EcorOBP20 | 790 | 138 | ref\|XP_005177698.1\| PREDICTED: general odorant-binding protein 69a [Musca domestica] | 3.00E-35 | 44% | 21 | Yes |
| Unigene11046 | EcorOBP21 | 437 | 137 | ref\|XP_011198820.1\| PREDICTED: general odorant-binding protein 28a-like [Bactrocera dorsalis] | 9.00E-34 | 43% | 18 | No |
| Unigene17716 | EcorOBP22 | 590 | 136 | ref\|NP_611442.1\| gb\|AAF57522.1\| Odorant-binding protein 56a [Drosophila melanogaster] | 5.00E-13 | 34% | 20 | Yes |
| Unigene28056 | EcorOBP23 | 450 | 136 | ref\|NP_611442.1\| gb\|AAF57522.1\| Odorant-binding protein 56a [Drosophila melanogaster] | 3.00E-15 | 38% | 18 | Yes |
| CL619.Contig1 | EcorOBP24 | 1505 | 135 | gb\|AID61309.1\| odorant binding protein [Calliphora stygia] | 4.00E-19 | 39% | 22 | Yes |
| CL1878.Contig1 | EcorOBP25 | 582 | 135 | ref\|NP_611444.2\| gb\|AAF57520.1\|Odorant-binding protein 56d, isoform A [Drosophila melanogaster] | 2.00E-11 | 27% | 20 | Yes |
| Unigene8647 | EcorOBP26 | 415 | 134 | ref\|XP_005188768.1\| PREDICTED: general odorant-binding protein 56a-like [Musca domestica] | 7.00E-08 | 30% | 19 | Yes |
| Unigene13343 | EcorOBP27 | 477 | 134 | ref\|XP_001361436.1\| gb\|EAL26014.1\|Odorant-binding protein 56a [Drosophila pseudoobscura pseudoobscura] | 4.00E-20 | 40% | 18 | Yes |
| Unigene7869 | EcorOBP28 | 598 | 132 | ref\|XP_001361436.1\| gb\|EAL26014.1\|Odorant-binding protein 56a [Drosophila pseudoobscura pseudoobscura] | 7.00E-15 | 34% | 18 | Yes |
| Unigene27965 | EcorOBP29 | 500 | 131 | dbj\|BAM78536.1\| odorant-binding protein [Phormia regina] | 4.00E-12 | 34% | NO | No |
| CL3545.Contig1 | EcorOBP30 | 666 | 130 | ref\|XP_001361436.1\| gb\|EAL26014.1\|Odorant-binding protein 56a [Drosophila pseudoobscura pseudoobscura] | 1.00E-16 | 39% | 20 | Yes |
| CL983.Contig3 | EcorOBP31 | 1935 | 130 | ref\|XP_001361436.1\| gb\|EAL26014.1\|Odorant-binding protein 56a [Drosophila pseudoobscura pseudoobscura] | 2.00E-17 | 38% | 18 | Yes |
| CL983.Contig5 | EcorOBP32 | 1818 | 130 | gb\|AID61309.1\| odorant binding protein [Calliphora stygia] | 2.00E-17 | 36% | 18 | Yes |
| Unigene11507 | EcorOBP33 | 477 | 129 | dbj\|BAM78536.1\| odorant-binding protein [Phormia regina] | 8.00E-16 | 35% | 18 | Yes |
| Unigene12165 | EcorOBP34 | 763 | 129 | dbj\|BAN59719.1\| odorant-binding protein [Delia antiqua] | 1.00E-08 | 25% | 18 | Yes |
| CL1878.Contig2 | EcorOBP35 | 371 | 122 | ref\|XP_005188768.1\| PREDICTED: general odorant-binding protein 56a-like [Musca domestica] | 5.00E-10 | 31% | NO | No |
| CL2290.Contig2 | EcorOBP36 | 380 | 114 | ref\|XP_011210425.1\| PREDICTED: general odorant-binding protein 99a-like [Bactrocera dorsalis] | 7.00E-08 | 32% | 16 | No |
| Unigene8588 | EcorOBP37 | 338 | 112 | gb\|ADG96056.1\| putative odorant binding protein [Stomoxys calcitrans] | 8.00E-07 | 36% | 22 | No |
| Unigene17465 | EcorOBP38 | 337 | 108 | ref\|XP_011184805.1\| PREDICTED: general odorant-binding protein 84a-like [Bactrocera cucurbitae] | 8.00E-25 | 54% | 24 | No |
| Unigene4558 | EcorOBP39 | 363 | 105 | ref\|XP_011189397.1\| PREDICTED: putative odorant-binding protein A5 [Bactrocera cucurbitae] | 5.00E-22 | 49% | 22 | No |
| Unigene27632 | EcorOBP40 | 297 | 98 | ref\|XP_001361436.1\| gb\|EAL26014.1\|Odorant-binding protein 56a [Drosophila pseudoobscura pseudoobscura] | 4.00E-17 | 40% | NO | No |
| CL3115.Contig2 | EcorOBP41 | 210 | 70 | gb\|AIE43941.1\| gb\|AIE43949.1\|odorant-binding protein 29, partial [Lutzomyia longipalpis] | 2.00E-08 | 50% | NO | No |
| CL657.Contig3 | EcorOBP42 | 268 | 64 | gb\|AID61296.1\| odorant binding protein [Calliphora stygia] | 1.00E-28 | 78% | NO | No |
| CL657.Contig4 | EcorOBP43 | 225 | 59 | ref\|XP_011187213.1\| PREDICTED: general odorant-binding protein 19a [Bactrocera cucurbitae] | 4.00E-12 | 54% | 23 | No |
| Unigene28188 | EcorOBP44 | 214 | 46 | gb\|AGM37951.1\| pheromone binding protein, partial [Hylamorpha elegans] | 2.00E-07 | 41% | NO | No |

Table S2-5. Unigenes of candidate chemosensory proteins in *E. balteatus* and *E. corollae*

| Unigene reference | Gene name | Length (nt) | ORF (aa) | Blastx best hit (Reference/Name/Species) | Evalue | Identity | Signal peptide | Full length |
| --- | --- | --- | --- | --- | --- | --- | --- | --- |
| ***E. balteatus*** |  |  |  |  |  |  |  |  |
| CL286.Contig2 | EbalCSP1 | 1963 | 321 | gb\|AIW65104.1\| chemosensory protein [Helicoverpa armigera] | 2.00E-37 | 40% | 20 | Yes |
| CL286.Contig1 | EbalCSP2 | 1661 | 213 | ref\|NP_001037069.1\| gb\|ABH88202.1\|chemosensory protein 9 precursor [Bombyx mori] | 2.00E-34 | 46% | 20 | Yes |
| Unigene10704 | EbalCSP3 | 569 | 142 | emb\|CBA11328.1\| chemosensory protein 2 [Glossina morsitans morsitans] | 5.00E-54 | 59% | 19 | Yes |
| CL1477.Contig2 | EbalCSP4 | 1736 | 127 | gb\|ACO83220.1\| putative chemosensory binding protein [Stomoxys calcitrans] | 3.00E-52 | 66% | 18 | Yes |
| Unigene13284 | EbalCSP5 | 448 | 112 | gb\|ADG96051.1\| putative chemosensory binding protein [Stomoxys calcitrans] | 1.00E-50 | 76% | 26 | Yes |
| Unigene4287 | EbalCSP6 | 260 | 86 | gb\|AID61322.1\| chemosensory protein [Calliphora stygia] | 7.00E-34 | 68% | No | No |
| ***E. corollae*** |  |  |  |  |  |  |  |  |
| CL1349.Contig6 | EcorCSP1 | 2035 | 361 | gb\|AJP61958.1\| chemosensory protein [Phenacoccus solenopsis] | 2.00E-32 | 58% | 20 | Yes |
| CL1349.Contig3 | EcorCSP2 | 2074 | 313 | gb\|AJP61958.1\| chemosensory protein [Phenacoccus solenopsis] | 2.00E-32 | 58% | 20 | Yes |
| CL1349.Contig1 | EcorCSP3 | 1646 | 219 | gb\|AJP61958.1\| chemosensory protein [Phenacoccus solenopsis] | 8.00E-33 | 58% | 20 | Yes |
| CL5306.Contig1 | EcorCSP4 | 599 | 140 | gb\|AID61325.1\| chemosensory protein [Calliphora stygia] | 9.00E-50 | 73% | 19 | Yes |
| CL3160.Contig1 | EcorCSP5 | 2680 | 127 | gb\|AID61324.1\| chemosensory protein [Calliphora stygia] | 8.00E-52 | 64% | 18 | Yes |
| Unigene5411 | EcorCSP6 | 534 | 113 | gb\|AID61322.1\| chemosensory protein [Calliphora stygia] | 2.00E-38 | 67% | 19 | No |
| Unigene18129 | EcorCSP7 | 902 | 112 | gb\|ADG96051.1\| putative chemosensory binding protein [Stomoxys calcitrans] | 2.00E-50 | 75% | 26 | Yes |

Table S2-6. Unigenes of candidate sensory neuron membrane proteins in *E. balteatus* and *E. corollae*

| Unigene reference | Gene name | Length (nt) | ORF (aa) | Blastx best hit (Reference/Name/Species) | Evalue | Identity | TMD (No.) | Full length |
| --- | --- | --- | --- | --- | --- | --- | --- | --- |
| ***E. balteatus*** |  |  |  |  |  |  |  |  |
| Unigene6502 | EbalSNMP1 | 2208 | 527 | ref\|XP_004519545.1\| PREDICTED: sensory neuron membrane protein 1 [Ceratitis capitata] | 0 | 69% | 1 | Yes |
| CL2542.Contig1 | EbalSNMP2 | 1862 | 557 | ref\|XP_004525758.2\| PREDICTED: sensory neuron membrane protein 2 isoform X2 [Ceratitis capitata] | 0 | 72% | 2 | Yes |
| ***E. corollae*** |  |  |  |  |  |  |  |  |
| CL1622.Contig1 | EcorSNMP1 | 2052 | 527 | ref\|XP_004519545.1\| PREDICTED: sensory neuron membrane protein 1 [Ceratitis capitata] | 0 | 67% | 2 | Yes |
| CL6519.Contig1 | EcorSNMP2 | 2177 | 557 | ref\|XP_011207383.1\| PREDICTED: sensory neuron membrane protein 2 isoform X2 [Bactrocera dorsalis] | 0 | 70% | 2 | Yes |
