# Supplementary material for: Chemosensory genes in the antennal transcriptome of two syrphid species,Episyrphus balteatusandEupeodes corollae (Diptera: Syrphidae)
Source: BMC Genomics. 2017 Aug 7;18:586. doi: 10.1186/s12864-017-3939-4 (PMC5547493; doi:10.1186/s12864-017-3939-4)
Supplement: Supplementary file 4 — Comparison of homologous ORs in E. balteatus and E. corollae. (DOCX 61 kb) [file 12864_2017_3939_MOESM4_ESM.docx]

Table S3. Comparasion of homologous ORs in *E. balteatus* and *E. corollae*

| Number | Ebal | Ecor | Identity | Full length |
| --- | --- | --- | --- | --- |
| 1 | Orco | Orco | 97.27 | Yes |
| 2 | OR29 | OR36 | 94.88 | Yes |
| 3 | OR3 | OR5 | 92.2 | Yes |
| 4 | OR13 | OR19 | 91.46 | Yes |
| 5 | OR1 | OR22 | 91.3 | Yes |
| 6 | OR5 | OR7 | 88.14 | Yes |
| 7 | OR21 | OR28 | 87.6 | Yes |
| 8 | OR2 | OR4 | 87.29 | Yes |
| 9 | OR25 | OR31 | 86.13 | No |
| 10 | OR8 | OR14 | 85.57 | Yes |
| 11 | OR33 | OR35 | 84.95 | No |
| 12 | OR14 | OR13 | 83.37 | No |
| 13 | OR7 | OR9 | 83.21 | Yes |
| 14 | OR11 | OR3 | 81.56 | Yes |
| 15 | OR23 | OR23 | 80.56 | No |
| 16 | OR27 | OR30 | 79 | No |
| 17 | OR12 | OR18 | 77.69 | Yes |
| 18 | OR28 | OR26 | 75.13 | No |
| 19 | OR19 | OR2 | 74.7 | Yes |
| 20 | OR4 | OR17 | 73.99 | No |
| 21 | OR18 | OR29 | 73.97 | No |
| 22 | OR16 | OR24 | 71.65 | Yes |
| 23 | OR22 | OR8 | 71.43 | No |
| 24 | OR10 | OR16 | 70.82 | Yes |
| 25 | OR17 | OR25 | 68.88 | No |
| 26 | OR20 | OR27 | 68.65 | Yes |
| 27 | OR42 | OR40 | 65.4 | No |
| 28 | OR34 | OR37 | 61.32 | No |
| 29 | OR31 | OR33 | 50.26 | No |
| 30 | OR36 | OR1 | 49.89 | No |
| 31 | OR40 | OR20 | 38.29 | No |
| 32 | OR39 | OR6 | 31.88 | No |
| 33 | OR47 | OR15 | 19.11 | No |
