# Supplementary material for: Chemosensory genes in the antennal transcriptome of two syrphid species,Episyrphus balteatusandEupeodes corollae (Diptera: Syrphidae)
Source: BMC Genomics. 2017 Aug 7;18:586. doi: 10.1186/s12864-017-3939-4 (PMC5547493; doi:10.1186/s12864-017-3939-4)
Supplement: Supplementary file 9 — Primers of candidate ORs in E. balteatus and E. corollae used for RT-PCR. (DOCX 20 kb) [file 12864_2017_3939_MOESM9_ESM.docx]

Table S5. Primers used for RT-PCR

| Primer | Sequences (5'to3') | Primer | Sequences (5'to3') |
| --- | --- | --- | --- |
| EbalOrco-F | CCAACTCCAAGTCAGAGCTTAT | EcorOrco-F | TTGGGTCGAACGGCATAAA |
| EbalOrco-R | GCTAGGGCATAGCTCAAGTATC | EcorOrco-R | GCACCAGCACCATGAAATAAG |
| EbalOR1-F | CTTGCCGGATGGAGTACTTATT | EcorOR1-F | GATCTGCCAAGGGAAGAACTTA |
| EbalOR1-R | GCTGGCCAAGAGCAGATATTA | EcorOR1-R | CCGAAGATCCAATCCCTGAAA |
| EbalOR2-F | GGTCGTGGTATGTGGTGTTT | EcorOR2-F | AAGAGAAGCCGTCGATGTTATC |
| EbalOR2-R | TTCCAGCAAACGAGGTGAATA | EcorOR2-R | ATCGATGTTGCAGGACCTTAC |
| EbalOR3-F | CGGCAGAAGATTGCTTGATTG | EcorOR3-F | CTTATACCCGTTGGGCAATCT |
| EbalOR3-R | CCCAGTTGCACATGTAGTATGA | EcorOR3-R | TGGACTCGCATCATACCAATTAT |
| EbalOR4-F | GAAGAATGAGCCGTGAAGATTTG | EcorOR4-F | GCTGAGGATTCTTTGATTGGATTT |
| EbalOR4-R | GCAGCGACCTCTAACGAATTA | EcorOR4-R | GCCAGCAAGTGAGGACTATT |
| EbalOR5-F | CACTGTGCAGCAAGGTATCA | EcorOR5-F | CACAGGATGGGTTGGGATTT |
| EbalOR5-R | TAGCTTGGGTGTTGCCATAAA | EcorOR5-R | TTTCAGGGTTTCCACACTAAGG |
| EbalOR6-F | CTATGGCACAACGGTTGAAATG | EcorOR6-F | TAGCTCCATCTGTTGCTTTGAG |
| EbalOR6-R | GCTCGTGTAAATTGCGTTACTG | EcorOR6-R | GCTCTCACTTATGATCGCTTGT |
| EbalOR7-F | GCCAGCATTGAATTGGGAATAG | EcorOR7-F | GGTCATTTCTGCTCACTCTCTT |
| EbalOR7-R | AGCGGCAATGATGGACTAAA | EcorOR7-R | GCTAAGGCCGTTCCTTTGTA |
| EbalOR8-F | TGCTGGGTTCTTCAGTCATC | EcorOR8-F | TCGCAGGCGACTTATTCTTC |
| EbalOR8-R | ATTGCTCGGTCACCACTTT | EcorOR8-R | CGGGATCTTGCGACTTCATTA |
| EbalOR9-F | GGCAATTACGCTGGAGACTTAC | EcorOR9-F | GTACACTGTCCAGCCGATTATAG |
| EbalOR9-R | GCCCATCCCGCAATAAAGATATAG | EcorOR9-R | CATCGCCATTACTCTCTGGTT |
| EbalOR10-F | TTCAAGCTGCTTTACCCTATCA | EcorOR10-F | CTGGAACTGATGGGACATTCTT |
| EbalOR10-R | TTAACACTCTCCGCTCCATAAC | EcorOR10-R | TGTAACGTATTCGGCACCATAG |
| EbalOR11-F | ACACCCTCAACGACTTTATGG | EcorOR11-F | GCACTTTGTCCGTGCTTAAC |
| EbalOR11-R | CGACGACAGCAACGATGTAT | EcorOR11-R | GATCCCATCAGCTCCTGATATTC |
| EbalOR12-F | CAGTGGTCCGGATTACCATTAT | EcorOR12-F | CAGTTGAAGCAGCCATTGTATTT |
| EbalOR12-R | ACTGACGTTATCGCTGGTATG | EcorOR12-R | GCAGCAGTCGAGTATTGTAGAG |
| EbalOR13-F | CAGCAGATGGTAACGAGGATAAA | EcorOR13-F | GGCACGGACAGAGATCTTATAG |
| EbalOR13-R | GGTCCCACCGATGCAATATAA | EcorOR13-R | TGTGAACGAGCCAACAATAAAG |
| EbalOR14-F | CTGGAACGGACGAAGATCTTATAG | EcorOR14-F | CTCTGACCACTGACAAAGAGTC |
| EbalOR14-R | ACGAGCTAACAGCAATGGTAA | EcorOR14-R | AGTTCTTGCACACTCCGATAC |
| EbalOR15-F | AGTCCATCCGAACAATGTCTAC | EcorOR15-F | ACTCTCACCGGGTGTAACTA |
| EbalOR15-R | CAGGCCACGTTAAGAAGATAGT | EcorOR15-R | GGGCTGCTCTATAGGGTAAATC |
| EbalOR16-F | TGCCCATTCGACGTGTATTT | EcorOR16-F | GCTGCTTTACCCTATGCAGATA |
| EbalOR16-R | TCTGAGCCTTCATCAGCATTATC | EcorOR16-R | TGCTCCGCTCCATAACAATAAG |
| EbalOR17-F | TTCTTTGGGTAGCAGCCTATAC | EcorOR17-F | TTTGGATCTACGGCCATTCG |
| EbalOR17-R | CACAAACATCCCACAGCAATAA | EcorOR17-R | CCCAAATCCTCACGACTCATT |
| EbalOR18-F | GGAGCTAAGTGAAATGGCTACT | EcorOR18-F | GTTCGGACTGCCATTGTTTG |
| EbalOR18-R | CTGAGCCTTCATCAGCATGTA | EcorOR18-R | GCTCTCAAACTCCATCCAAGA |
| EbalOR19-F | AGTTACAAGGGAAGCAGTTGAT | EcorOR19-F | CTCTGGCGTCATGACAGTATTT |
| EbalOR19-R | CAGTCATAAGTCCAAGTGCCATA | EcorOR19-R | CACCAATCCGATGCCTGTATTA |
| EbalOR20-F | CTCAACAGAATGGGACACTCA | EcorOR20-F | CGACAAGGCGAGAGAACTAATC |
| EbalOR20-R | ACAGCACGATCCGCTATTT | EcorOR20-R | ACTTGGGTGTGCCTTCATAC |
| EbalOR21-F | GGACCCACTTTATCGTCTCTATTC | EcorOR21-F | CTTTGGGCTTGTGGCATATTT |
| EbalOR21-R | TACCAATTGCTACCACCGTAAA | EcorOR21-R | TGCTCAACTGACGAAACTAGAA |
| EbalOR22-F | TTGCCCGGATTGGATGTT | EcorOR22-F | CGTCAGCAACCACCTCTATT |
| EbalOR22-R | GTGTTGGTAGTCTTGGCATATTG | EcorOR22-R | CCAGTTGGACAATACCTCTCTATC |
| EbalOR23-F | TCGAGTGAGCGGACTCTAAT | EcorOR23-F | GGCTACCTTTGGATTGGAAGA |
| EbalOR23-R | GAGCTAGTCCAGTGCACATAAA | EcorOR23-R | GCACATGAACTGAGCGAAAC |
| EbalOR24-F | GAGCCTGATCAAACCAACAAAG | EcorOR24-F | CCGTTGCTAAGGGACATTCTAA |
| EbalOR24-R | GCGATCGCATTAGGACCATTA | EcorOR24-R | CGTTCTCCCACAGACAATCTATAC |
| EbalOR25-F | CCAACTGCCTTACACGATTTAC | EcorOR25-F | CTCTGCCGTCAATCTTGGATAA |
| EbalOR25-R | GGAAACCAAGCTGGATAGAGA | EcorOR25-R | ACCAATGAGAGACAGGAAACATAG |
| EbalOR26-F | GATTTCTGGGCCCGTATTGA | EcorOR26-F | TTAGCAGTGTCTGGCGTTATT |
| EbalOR26-R | TCGATCCTTGAAGTCACAAACA | EcorOR26-R | CCGACAGGATCTTCTTCAACTC |
| EbalOR27-F | TGATGGCGGGCCTAATTAATAC | EcorOR27-F | GTCGTGTCAGCTCAATATCCAA |
| EbalOR27-R | ATCCACCAGAGACAACACAAG | EcorOR27-R | GCCAACGGAGTCAGAAGAAA |
| EbalOR28-F | GTACTACCCTCGCAACTTATGG | EcorOR28-F | GCAGCCGATTCGAGGAAATA |
| EbalOR28-R | CCGAAATAACAGGGCTCAAGTA | EcorOR28-R | TGTCACAGTCAGGCCAATAAG |
| EbalOR29-F | GTTCCAGGAGTCAACGAATACA | EcorOR29-F | GCCGTATTGTATGCTGTTGTG |
| EbalOR29-R | TCTGAGCACGTAAGATCAACAG | EcorOR29-R | ACCTTCTCGTCTTTGCTATCG |
| EbalOR30-F | CCTGGCTCATACTGGAACAATAA | EcorOR30-F | TGAGGCTGCTAAAGATGAAGAA |
| EbalOR30-R | CCAATGTGATGCTCGCAAATG | EcorOR30-R | CCGAAATACCAGCAATGTGAAG |
| EbalOR31-F | TCCAGAGAGATCCGTGAAATTG | EcorOR31-F | GCATCCTCACAGGTCATCTTAG |
| EbalOR31-R | TCCGAATGAAGTTGTTGACTCT | EcorOR31-R | ACCGCACAAGCAGAGTAAA |
| EbalOR32-F | ACTTGGGTTTCTTTGCCATTTC | EcorOR32-F | TGGCTTATCCTGTCTGCATTTA |
| EbalOR32-R | CATTCCTGGAGCCAATCGAATA | EcorOR32-R | ATCTCAGGGTTTCCACCATTT |
| EbalOR33-F | GGGCCAAGTGGATACGTTTAT | EcorOR33-F | TGCCATCCAAGCCATTCA |
| EbalOR33-R | CAACTTCCTAAGCCTCATCTGG | EcorOR33-R | CCGCAGTACACCGTATCATATC |
| EbalOR34-F | TCGCTAACATCGCTGGTAAAT | EcorOR34-F | CTGGCTGTGATGGAACCTTAT |
| EbalOR34-R | GTCTCAAGTGTCATTGGGTACA | EcorOR34-R | AACCCTTTCAGCTCCATAGC |
| EbalOR35-F | AGCCAGTCATGTTGTGGTATG | EcorOR35-F | TCCTTGCTACGAACTGTAATGG |
| EbalOR35-R | AAGTCGCCAAGCAATTGAAATC | EcorOR35-R | CGATGTGAGGATGACAGGAAAT |
| EbalOR36-F | TGTGGGAATGCTTATCTTGGT | EcorOR36-F | AGCCTACTTGACGAAACTAACC |
| EbalOR36-R | AATAAGCCAAAGCGAATTGATAGG | EcorOR36-R | AGAAACAGCAGGGCACATAA |
| EbalOR37-F | TGCCACTATTGCTCTGCTATC | EcorOR37-F | TTGTCACCTACATACCGTTCAC |
| EbalOR37-R | CTGGCCAAGCTCCAGTAAAT | EcorOR37-R | TCATTGGGTACATTCCACCAG |
| EbalOR38-F | CGGTTGATGGGTTTCGAATATG | EcorOR38-F | GTACACTGTCCAGCCGATTATAG |
| EbalOR38-R | CATACCACACCTTCCAAGGATAA | EcorOR38-R | TCATCGCCATTACTCTCTGATTT |
| EbalOR39-F | GCCTTACCCAGCGCTTTAT | EcorOR39-F | GGTCTGGAATGCCCTTGTT |
| EbalOR39-R | TATCCTTTCCCAAGCCAACAG | EcorOR39-R | CGTCCCGCTGTTCTTTACTT |
| EbalOR40-F | CCAGGCAGGAAGAAGAATCTAA | EcorOR40-F | GCCCAGGAACCATGGAAATA |
| EbalOR40-R | TGTACTTTGGTGTGCCTTCATA | EcorOR40-R | CCCAGTTACACGAGTAGTATGC |
| EbalOR41-F | TATTGCTGCCTGGCTCATAC | EcorOR41-F | GATGCTTCAGGTCTTGTTATGATTT |
| EbalOR41-R | CCAATGTGATGCTCGCAAATAC | EcorOR41-R | AGTAGGGCCACAACAGAATATG |
| EbalOR42-F | CTGCCGAGGATTGTTTGATTG | EcorRPS3-F | TCGTGAACTCGCAGAAGATG |
| EbalOR42-R | TCACCATAAGCGAGAAGTGATT | EcorRPS3-R | TGAAACGGAGCACACCATAG |
| EbalOR43-F | TCGTGAAACACATCCTGTATCG |  |  |
| EbalOR43-R | AGTCGCGATTTCCTTGAGTAAT |  |  |
| EbalOR44-F | TTGGAACTTCTTTGCACTTCTT |  |  |
| EbalOR44-R | CCTGTCTTAAACGATCTGCATTC |  |  |
| EbalOR45-F | TTAAACCTACCACAGCCAAGG |  |  |
| EbalOR45-R | AAACCAATCGTAGACTTCTCTTACA |  |  |
| EbalOR46-F | CGTTCCGACTCATTGGAAGT |  |  |
| EbalOR46-R | TTGCGTAGTAAGAACCCTTGG |  |  |
| EbalOR47-F | GGCTCTGCCCTATGAAGATATG |  |  |
| EbalOR47-R | CCAAAGTAATGCTCCCGATCT |  |  |
| EbalOR48-F | TCGAGAGGCTTGAATCTTGAAA |  |  |
| EbalOR48-R | GTTAACCGATTCCGATGCAAAG |  |  |
| EbalOR49-F | AGCAATACAATATTCAAAGGAGACC |  |  |
| EbalOR49-R | GCCAGGGTGATCTGAGTAAAG |  |  |
| EbalOR50-F | TAACCTGGGATGTGTGGTATG |  |  |
| EbalOR50-R | CTTTGATGAGCACCGATTTCC |  |  |
| EbalRPS3-F | GGTTGCGAAGTTGTCGTTTC |  |  |
| EbalRPS3-R | CCTCGCTCCCTTTACCATTT |  |  |
